# Public health impact and cost-effectiveness of the RTS,S/AS01 malaria vaccine: a systematic comparison of predictions from four mathematical models

**DOI:** 10.1016/S0140-6736(15)00725-4

**Published:** 2016-01-23

**Authors:** Melissa A Penny, Robert Verity, Caitlin A Bever, Christophe Sauboin, Katya Galactionova, Stefan Flasche, Michael T White, Edward A Wenger, Nicolas Van de Velde, Peter Pemberton-Ross, Jamie T Griffin, Thomas A Smith, Philip A Eckhoff, Farzana Muhib, Mark Jit, Azra C Ghani

**Affiliations:** a Department of Epidemiology and Public Health, Swiss Tropical and Public Health Institute, Basel, Switzerland; b University of Basel, Basel, Switzerland; c Medical Research Council Centre for Outbreak Analysis and Modelling, Imperial College London, London, UK; d Institute for Disease Modeling, Bellevue, WA, USA; e GSK Vaccines, Wavre, Belgium; f Department of Infectious Disease Epidemiology, London School of Hygiene & Tropical Medicine, London, UK; g PATH, Washington, DC, USA; h Modelling and Economics Unit, Public Health England, London, UK

## Abstract

**Background:**

The phase 3 trial of the RTS,S/AS01 malaria vaccine candidate showed modest efficacy of the vaccine against *Plasmodium falciparum* malaria, but was not powered to assess mortality endpoints. Impact projections and cost-effectiveness estimates for longer timeframes than the trial follow-up and across a range of settings are needed to inform policy recommendations. We aimed to assess the public health impact and cost-effectiveness of routine use of the RTS,S/AS01 vaccine in African settings.

**Methods:**

We compared four malaria transmission models and their predictions to assess vaccine cost-effectiveness and impact. We used trial data for follow-up of 32 months or longer to parameterise vaccine protection in the group aged 5–17 months. Estimates of cases, deaths, and disability-adjusted life-years (DALYs) averted were calculated over a 15 year time horizon for a range of levels of *Plasmodium falciparum* parasite prevalence in 2–10 year olds (*Pf*PR_2–10_; range 3–65%). We considered two vaccine schedules: three doses at ages 6, 7·5, and 9 months (three-dose schedule, 90% coverage) and including a fourth dose at age 27 months (four-dose schedule, 72% coverage). We estimated cost-effectiveness in the presence of existing malaria interventions for vaccine prices of US$2–10 per dose.

**Findings:**

In regions with a *Pf*PR_2–10_ of 10–65%, RTS,S/AS01 is predicted to avert a median of 93 940 (range 20 490–126 540) clinical cases and 394 (127–708) deaths for the three-dose schedule, or 116 480 (31 450–160 410) clinical cases and 484 (189–859) deaths for the four-dose schedule, per 100 000 fully vaccinated children. A positive impact is also predicted at a *Pf*PR_2–10_ of 5–10%, but there is little impact at a prevalence of lower than 3%. At $5 per dose and a *Pf*PR_2–10_ of 10–65%, we estimated a median incremental cost-effectiveness ratio compared with current interventions of $30 (range 18–211) per clinical case averted and $80 (44–279) per DALY averted for the three-dose schedule, and of $25 (16–222) and $87 (48–244), respectively, for the four-dose schedule. Higher ICERs were estimated at low *Pf*PR_2–10_ levels.

**Interpretation:**

We predict a significant public health impact and high cost-effectiveness of the RTS,S/AS01 vaccine across a wide range of settings. Decisions about implementation will need to consider levels of malaria burden, the cost-effectiveness and coverage of other malaria interventions, health priorities, financing, and the capacity of the health system to deliver the vaccine.

**Funding:**

PATH Malaria Vaccine Initiative; Bill & Melinda Gates Foundation; Global Good Fund; Medical Research Council; UK Department for International Development; GAVI, the Vaccine Alliance; WHO.

## Introduction

In the past 15 years, renewed investment in malaria control has led to substantial reductions in malaria worldwide.^1^, ^2^ Despite this investment, malaria remains a leading cause of morbidity and mortality.^1^ Investigators have completed phase 3 testing of the RTS,S/AS01 *Plasmodium falciparum* vaccine candidate in two age groups at 11 centres in sub-Saharan Africa.^3^ Efficacy against clinical malaria was 20·3% (95% CI 13·6–26·5) in infants aged 6–12 weeks and 35·2% (30·5–39·5) in children aged 5–17 months during 32 months of follow-up. Vaccine efficacy against clinical malaria declined over time, from 45·1% (95% CI 41·4–48·7%) during months 0–20 to 16·1% (8·5–23·0%) during months 21–32 in children, and from 27·0% (21·1–32·5) to 7·6% (−1·4 to 15·9%), respectively, in infants. In the group given a fourth dose of vaccine 18 months after the initial course, efficacy against clinical malaria was 43·9% (95% CI 39·7–47·8) in children and 27·8% (21·7–33·4) in infants over 32 months.

Levels of malaria incidence and seasonality profiles varied across the 11 trial sites, and malaria incidence ranged from 0·03 to 4·27 clinical episodes per infant per year in the control group, which is broadly representative of settings in Africa.^4^ However, these estimates were obtained in cohorts with a high coverage of long-lasting insecticide-treated nets (75–80% in both intervention and control groups) and in the presence of high levels of good-quality access to care. Thus, mortality was lower in children in the control group than in the general population outside the trial and elsewhere in Africa, as shown for one of the study sites.^5^ Of note was the very small number of malaria deaths (19 [<1%] of 8922 children died over a median of 48·1 months [IQR 39–50] of follow-up^3^), probably because of the high level of care for trial participants. Estimates of the public health impact and cost-effectiveness of the vaccine in different African settings with more typical access to care and current intervention coverage are needed to inform global and national decisions about vaccine introduction. In particular, estimations should be made of the impact on both morbidity and mortality in the absence of high levels of treatment to ensure that appropriate comparisons can be made between the cost-effectiveness of this vaccine and other childhood vaccines.

**Research in context**

**Evidence before this study**

The phase 3 trial of the RTS,S/AS01 vaccine provides estimates of cases averted at the 11 trial sites. However, these estimates need to be translated into full population impact to calculate the cost-effectiveness of the vaccine compared with other malaria interventions. Mathematical models have previously estimated the public health impact and cost-effectiveness of malaria vaccines in different settings. We searched PubMed, the Cochrane Library, and other relevant data sources between April 1, 2015, and June 8, 2015, for studies of predictions and the cost-effectiveness of RTS,S malaria vaccine. The literature covered the period 2006, to June, 2015. We searched PubMed with the MeSH terms “RTS,S” [All Fields] AND “malaria” [All Fields] AND “model” [All Fields] OR “simulation” [All Fields] OR “prediction” [All Fields]. For the Cochrane Library and other data sources, we used the search terms “RTS,S”, AND “predictions”. The 29 manuscripts identified included 14 reports that referred to RTS,S or pre-erythrocytic vaccines and model analysis or predictions. Ten of these reports did not use trial data to parameterise models or were limited to data from phase 2 trials of RTS,S with a different adjuvant (AS02) or for vaccination in a different age group, whereas the other four reports made predictions of RTS,S or similar malaria vaccine candidates, but with vaccines parameterised from either intermediate phase 3 results or phase 2 trial data for RTS,S/AS01. Of these 14 publications, 12 were based on models included in the present study. A search for “malaria model” [All Fields] and “comparison” [All Fields] identified no publications that compared malaria transmission models.

**Added value of this study**

This is the first study to estimate the public health impact of the vaccine and its cost-effectiveness in populations beyond the trial with multiple mathematical models to address structural model uncertainty. This is also the first modelling study to use final site-specific results of the RTS,S phase 3 clinical trial and to systematically compare the malaria transmission models. Predictions are made for the full range of *Plasmodium falciparum* parasite prevalence settings in Africa.

**Implications of all available evidence**

The RTS,S malaria vaccine candidate provided modest protection against clinical malaria in children across different disease burden settings in the phase 3 clinical trial. A WHO recommendation for vaccine use in endemic Africa will additionally need to consider the public health impact and cost-effectiveness informed by our model predictions. This study reports on the major collaborative exercise coordinated by WHO undertaken for this purpose. Our results quantify the potential number of cases and deaths that could be averted by the RTS,S/AS01 vaccine when implemented in moderate to high transmission with full vaccine coverage and in the presence of existing malaria control interventions. Decisions about implementation will need to consider levels of malaria burden, the cost-effectiveness and coverage of other malaria interventions, health priorities, financing, and the capacity of the health system to deliver the vaccine.

Mathematical models of malaria dynamics can be useful for estimation of the potential public health impacts of malaria vaccination beyond the efficacy estimates obtained from the individually randomised clinical trial. An important feature of these models is that they have been extensively parameterised to routine data from endemic settings. Additionally, the models capture potential age-shifting of cases to older ages, as shown for severe malaria in the phase 3 trial, but that would not be expected to be shown in 4 years of follow-up. Furthermore, via use of data relating clinical disease to death, models can be used to estimate effects on mortality based on realistic assumptions about treatment in non-trial settings. In line with WHO recommendations about use of cost-effectiveness information within the decision-making process for introduction of new vaccines,^6^, ^7^ and for impact estimates from GAVI, the Vaccine Alliance,^8^ we systematically compared four malaria transmission models to assess the public health impact and cost-effectiveness of routine use of the RTS,S/AS01 vaccine in African settings.

## Methods

### Models and harmonisation assumptions

Modelling groups were contacted by WHO to participate in this study. Four groups agreed to contribute: the Institute for Disease Modeling (EMOD-DTK),^9^ GSK Vaccines (GSK),^10^ Imperial College London (Imperial),^11^ and the Swiss Tropical and Public Health Institute (OpenMalaria).^12^ The four models encompass a range of structures and levels of complexity, with all simulating malaria transmission and vaccine impact for defined geographic areas, taking into account local exposure and population demographics. Two models (Imperial and OpenMalaria) capture any herd effect of vaccination, whereas the other two models (EMOD-DTK and GSK) are implemented as a fixed-exposure cohort. Appendix pp 5–20 provide a full comparison of the models.

We used trial data^3^ for follow-up of 32 months or longer to parameterise vaccine efficacy against infection for the group aged 5–17 months. EMOD-DTK, GSK, and OpenMalaria^13^ used site-specific incidence or efficacy data aggregated into 3 month periods, and Imperial^14^ used individual-level data. We assumed no partial protection from the initial two doses, consistent with primary reporting from the trial.^3^ With use of these estimates derived from the trial data, we then made projections for wider transmission settings representative of normal access to care. Demographics, *Plasmodium falciparum* parasite prevalence in 2–10 year olds (*Pf*PR_2–10_; a standard metric used to describe the intensity of malaria transmission^2^), seasonality, and case management were aligned across the models (table 1). We assumed that access to artemisinin-based combination therapy was 45%, at the top of the range reported in 2013,^1^ but significantly lower than in the trial.

We predicted the public health impact and cost-effectiveness of the vaccine for a range of *Pf*PR_2–10_ levels (3–65%), assuming vaccine implementation was in addition to existing levels of malaria control interventions and treatment under the assumptions in table 1. We considered two immunisation schedules: three doses at age 6, 7·5, and 9 months (6–9 months with a three-dose schedule), and with an additional fourth dose at 27 months of age (6–9 months with a four-dose schedule). We chose these schedules because two visits (months 6 and 9) align with routine health-care visits (vitamin A supplement at 6 months and measles complex vaccine at 9 months). We focus on the cohort aged 5–17 months because efficacy was higher in this group than in the group aged 6–12 weeks.^3^ For both schedules, we defined a fully vaccinated child as a child who had received at least three doses.

We assumed 90% vaccine coverage (similar to diphtheria-tetanus-pertussis [DTP3] in Africa^16^) for 6–9 month implementation, with 80% of these individuals receiving the fourth dose (72% coverage) based on a drop-off in coverage from DTP3 to measles vaccination at 9 months (table 1).^16^ We summarise outputs as clinical cases, severe cases, deaths, and undiscounted disability-adjusted life-years (DALYs; driven mainly by mortality) averted per 100 000 fully vaccinated children. Outputs are cumulative over a 15 year time horizon, chosen to be comparable to the timeframe of the new Global Technical Strategy for malaria^17^ and over a length of time that is long enough to capture any potential detrimental effect of shifting of cases to older ages. We report health outcomes for the entire population and for children younger than 5 years.

We did this study in accordance with Good Clinical Practice guidelines and the Declaration of Helsinki. The trial protocol was approved by the ethical review board at each study centre and partner institution and by the national regulatory authority in each country.^18^ Because this work involves data simulations and analysis, informed consent was not required.

### Statistical analysis

We estimated the incremental cost per clinical case or DALY averted compared with no vaccination with standard levels of access to treatment and other malaria interventions (with costs discounted at 3%). We evaluated incremental cost for assumed vaccine purchase prices of US$2, $5 and $10, corresponding to a cost per dose of $2·69, $6·52, and $12·91, respectively (table 1, appendix pp 33–34). We calculated costs from the perspective of the health-care provider, which include the cost of consumables (vaccines and immunisation supplies) for RTS,S introduction, diagnostics, and antimalarial drugs, and related commodities for malaria case management (table 1). We undertook a univariate sensitivity analysis of vaccine properties, health systems, and economic parameters for the OpenMalaria model (appendix pp 48–50).

We present outputs from each model as medians and 95% prediction intervals. We present summary outputs across the four models as the median of all models plus ranges across the medians of the individual models.

### Role of the funding sources

The PATH Malaria Vaccine Initiative contributed to study design and data interpretation. The Health Economics Group of GSK Vaccines was involved in data analysis, data interpretation, and writing of the report (as one model group in the comparison), but had no role in the opinions and results presented by the other groups. All other funders of the study had no role in study design, data analysis, data interpretation, or writing of the report. All authors had full access to the data and were jointly responsible for the decision to submit the manuscript.

## Results

**Figure 1:**
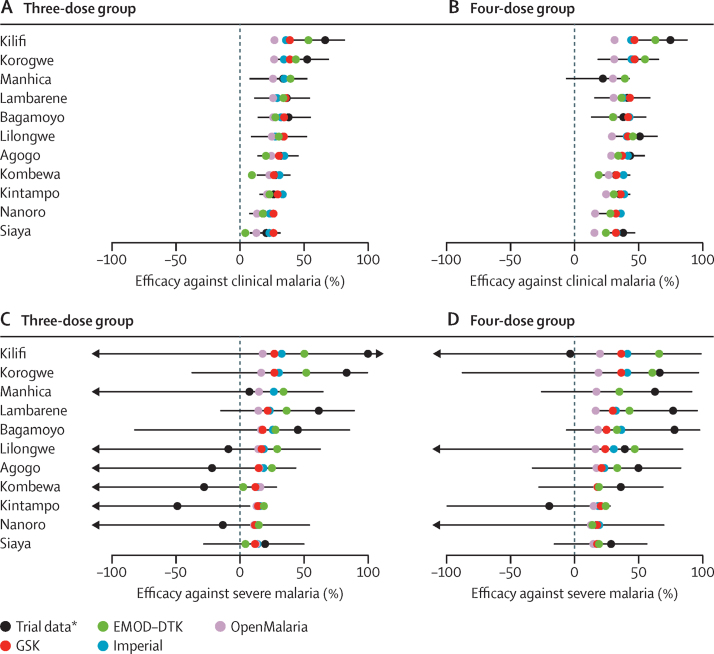
Observed and model-predicted vaccine efficacy against clinical and severe malaria from month 0 to study end (≥32 months) by study site in the 5–17 month age category Vaccine efficacy against all episodes of clinical malaria (primary case definition) in the three-dose group (A) and the four-dose group (B), and against severe malaria (primary case definition) in the three-dose group (C) and the four-dose group (D). Error bars show 95% CIs estimated from the trial data. *Intention-to-treat analysis.

Figure 1 shows the trial and model-estimated cumulative vaccine efficacy by trial site at study end (≥32 months). Model-estimated efficacy generally falls within the confidence intervals of the trial data for both clinical malaria and severe disease (figure 1). The estimated underlying protection against infection was similar across models during the first 18 months, but diverged as the projections extended beyond the trial period (appendix pp 21–31).

**Figure 2:**
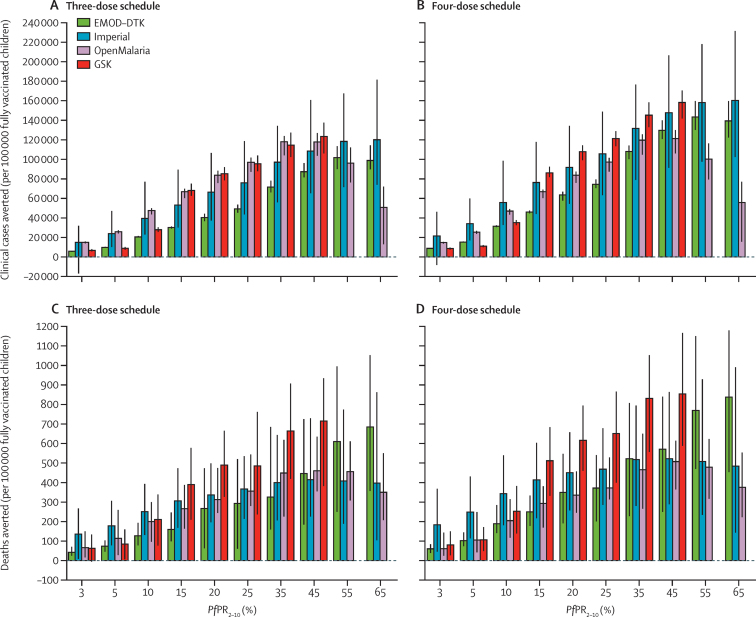
Model predictions of clinical cases and deaths averted per 100 000 children fully vaccinated with a three-dose or four-dose immunisation schedule for a range of baseline *Pf*PR_2–10_ levels Results for the three-dose (A, C) and four-dose (B, D) schedules are cumulative following 15 years of routine use of RTS,S. Bars show median estimates and error bars show 95% credible intervals. Negative cases averted at low transmission are due to stochastic variation between model runs at low prevalence, rather than to any modelled biological mechanism. *Pf*PR_2–10_=parasite prevalence in 2–10 year olds.

**Figure 3:**
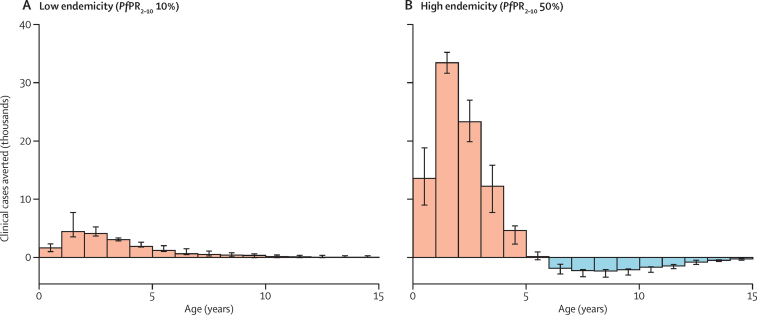
Clinical cases averted in the vaccinated cohort at low (A) and high (B) endemicity after 15 years of routine use of RTS,S in a four-dose immunisation schedule Bars show median predictions over all four models and error bars show the range of predictions. Uncertainty within each model is not shown (see appendix pp 5–7). *Pf*PR_2–10_=parasite prevalence in 2–10 year olds.

With the four mathematical models and our assumptions about implementation of the vaccine beyond the trial sites, we estimated that the absolute vaccine impact would increase with increasing levels of malaria transmission, averting from 15–32% of all clinical cases at a *Pf*PR_2–10_ of 10%, to 5–22% of clinical cases at a *Pf*PR_2–10_ of 65% (figure 2, table 2). Similarly, we estimated that 8–35% of malaria deaths in children younger than 5 years would be averted at a *Pf*PR_2–10_ of 10%, and 5–24% would be averted at a *Pf*PR_2–10_ of 65% (figure 2, table 2). The predicted lower proportion of events averted in higher versus lower transmission areas is partly a result of age-shifting of disease in higher transmission areas (figure 3, appendix pp 36–47). Across the models this finding translates to a median of 116 480 (range 31 450—160 410) cases of clinical malaria averted and 484 (189–859) deaths averted per 100 000 vaccinated children with the four-dose schedule for a *Pf*PR_2–10_ of 10–65% (figure 2, table 2). Public health impact at a *Pf*PR_2–10_ of less than 3% is expected to be small (figure 2, appendix pp 36–47).

There was a lack of consensus between the models for the additional public health impact of the four-dose versus the three-dose schedule. Three models (GSK, EMOD-DTK, Imperial) predicted a substantial additional impact of the four-dose schedule (16–43% extra deaths averted and 21–55% extra clinical cases averted; table 2), whereas the fourth model (OpenMalaria) predicted a marginal impact. This variance is due to differences in the estimated impact of the fourth dose against infection and the estimated waning of protection against infection between the models (appendix pp 21–31).

As expected with partially effective malaria control interventions,^19^, ^20^ we predicted a shift in cases to older ages due to reduced exposure and hence a delay in the development of naturally acquired immunity. Combined with the estimated waning of protection against infection of the vaccine, some of the initial impact of the vaccine on cases averted in very young children is therefore offset by predicted higher relative incidence at older ages (figure 3). This effect is predicted to be further delayed with the four-dose schedule (appendix pp 36–47). Similar effects are predicted for severe disease, with the age-shift occurring earlier than for clinical cases (appendix pp 36–47). Despite these findings, the overall cumulative impact of the vaccine on clinical cases, severe disease, and mortality over 15 years is predicted to be positive with the four-dose vaccine schedule (figure 3). Our analyses also assume that malaria exposure remains static for the length of follow-up. Thus, this age-shifting effect could be mitigated if transmission declines over this time horizon (eg, as a result of continued scale-up of other interventions^21^).

**Figure 4:**
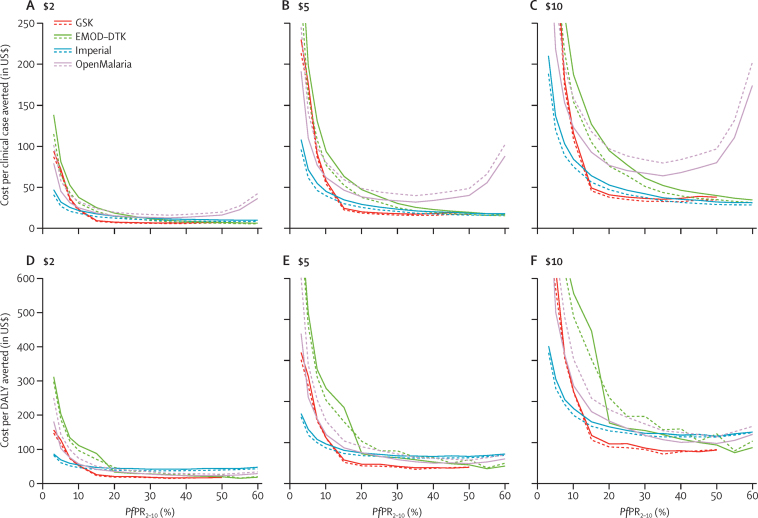
Cost per clinical case or DALY averted as a function of baseline parasite prevalence in 2–10 year olds (*Pf*PR_2–10_) Results assume a vaccine price of $2 (A, D), $5 (B, E), or $10 (C, E) per dose. Solid lines represent a three-dose immunisation schedule and dashed lines represent a four-dose immunisation schedule. Similar estimates of incremental cost-effectiveness ratios were obtained for the three-dose and four-dose schedules because the additional public health benefit of the boosted schedule is offset by the incremental cost of implementation of the additional dose. Uncertainty estimates surrounding the models' predictions are omitted for readability but overlap. DALY=disability-adjusted life-year.

The estimated incremental cost-effectiveness ratios (ICERs) for clinical cases and DALYs averted with both vaccination schedules compared with no vaccination were lowest at intermediate levels of *Pf*PR_2–10_, but were generally less than $100 per DALY averted for a *Pf*PR_2-10_ of more than 10% for a vaccine price of $5 per dose (table 2, figure 4). At a *Pf*PR_2–10_ of less than 10% the vaccine is estimated to become substantially less cost-effective due to fewer cases and deaths being averted for the same overall cost of a vaccine programme (figure 4). Furthermore, consensus between the models is lower at a *Pf*PR_2–10_ of less than 10% than at a *Pf*PR_2–10_ of more than 10% (figure 4). The predicted ICER of a four-dose schedule varied between the models because of the different public health impact projections of the fourth dose. However, overall we estimated that the ICERs (compared with no vaccination) for the four-dose schedule were similar to those estimated for the three-dose schedule (table 2). This similarity is because the extra deaths averted with the four-dose schedule are offset by the extra costs estimated for delivery of the fourth dose, and because of our assumption that only 80% of children who receive the first three doses will return for the fourth dose. In a sensitivity analysis, the cost per DALY averted over the 15 year time horizon at most doubled from the baseline estimate when we considered a range of factors including lower vaccination coverage, lower estimates of vaccine efficacy, and higher vaccine price, with the greatest impact due to a price increase from $5 to $10 (appendix pp 48–50).

## Discussion

We predict a positive public health impact of the introduction of RTS,S/AS01 in settings with a *Pf*PR_2–10_ between 3% and 65% over a 15 year time horizon with treatment levels representative of the current status in Africa. The absolute impact is substantial, with an estimated 116 500 (range 30 900–160 000) cases of clinical malaria and 484 (195–838) deaths averted per 100 000 vaccinated children with a four-dose schedule for a *Pf*PR_2–10_ between 10% and 65%. This finding translates to roughly one malaria death prevented for every 200 children fully vaccinated, which, at a vaccine price per dose of $5, equates to $87 (range 43–238) per DALY averted. WHO estimates that 528 000 (range 315 000–689 000) malaria deaths occurred in Africa in 2013.^1^ Depending on the area of implementation, we estimate that 6–30% of deaths in children younger than 5 years could potentially be averted by RTS,S, when added to existing coverage of long-lasting insecticide-treated nets (68%^1^, ^22^) and with moderate levels of malaria treatment.

There was no statistically significant impact against severe disease measured in the trial for the three-dose schedule (4·5%, 95% CI −20·6 to 24·5 at 32 months' follow-up in the group aged 5–17 months), although there was sustained significant efficacy with the four-dose schedule (32·2%, 13·7–46·9).^3^ Moreover, there was no statistically significant impact on malaria mortality and all-cause mortality, although numbers of deaths were small and the trial was not powered to assess this outcome.^3^ By contrast, we predicted a net positive impact on severe disease cases and hence deaths averted with the three-dose schedule, with an incremental benefit of the fourth dose of 22% extra deaths averted depending on the setting. There are several possible reasons for this discrepancy. First, severe disease incidence in the trial was low, with substantial differences between sites in the point estimates and wide confidence intervals. Second, inferences about severe disease made by the models are based mainly on data from sites with poorer quality of care than in the trial and broader case definitions.^23^ Third, the model projections assume that the fourth dose reaches only 80% of the third-dose recipients, and consequently only 72% of the eligible population receive four doses (compared with near 100% coverage in the trial). Finally, these projections are for a 15 year time horizon whereas the trial data include a maximum of 4 years of follow-up. Partially protective malaria interventions reduce an individual's exposure to malaria infection resulting in a delay in the acquisition of natural immunity^19^, ^20^ and a shift of clinical and severe disease to older ages.^19^, ^20^, ^21^ This shift is predicted to be greatest in scenarios with high initial vaccine impact. Much of the age-shift effect is predicted to happen beyond 3·5–4 years and at a *Pf*PR_2–10_ at the upper end of or higher than the *Pf*PR_2–10_ in the trial, and is therefore not expected to be shown in the trial. As such, longer-term follow-up of trial participants is needed to fully understand any shifting of cases to older age groups, with this shift partly shown in the 4 year follow-up of one of the phase 2 trials.^24^

For regions where *Pf*PR_2–10_ is more than 10%, RTS,S is predicted to be cost-effective compared with standard norms and thresholds. Broadly similar ICER estimates were obtained for the three-dose and four-dose schedules, with the additional public health benefit of the boosting schedule offset by the incremental cost of implementation of the additional dose. The higher cost of the four-dose versus the three-dose schedule is partly due to our assumption (based on the difference between DTP3 and first-dose measles vaccination coverage in Africa^16^) that only 80% of children who receive the first three doses would return for the fourth dose.^16^ At low prevalence (*Pf*PR_2–10_ 3%), when cases typically occur in older children and adults, we consistently noted that the vaccine was not cost-effective. Except for at very low prevalence, our estimated ICERs are lower than the national gross domestic products per person (median $842 [IQR 531–1668] across 43 malaria-endemic African countries with *Pf*PR_2–10_>10% in 2014^2^). Furthermore, in settings with *Pf*PR_2–10_ levels of 20% or greater, ICERs at a vaccine price of $5 are less than $100 per DALY averted. A 2011 review^25^ summarised average incremental costs per DALY averted (2009 prices) for long-lasting insecticide-treated nets of $27 (range 8·15–110), insecticide residual spraying of $143 (135–150), and intermittent preventative treatment of $24 (1·08–44·24). Additionally, economic assessment of long-lasting insecticide-treated nets from large-scale field studies^26^, ^27^ estimated that the cost per DALY averted was between $13 and $89. However, there was wide variation in the costing methodologies used and economies of scale captured by these studies; therefore, these figures should be interpreted as indicative ranges rather than directly corresponding to our estimates. With these caveats, RTS,S is somewhat less cost-effective than long-lasting insecticide-treated nets, which are regarded as one of the most cost-effective interventions available for malaria control; this is an important point for countries to bear in mind because use of long-lasting insecticide-treated nets has also led to proven reductions in all-cause mortality.^27^

Despite the differences between the models, all four groups reached consensus about the expected public health impact and cost-effectiveness of RTS,S/AS01 administration to children aged 6–9 months, and their consensus predictions help to define priority areas by levels of *Pf*PR_2–10_ for policy recommendation. The systematic and harmonised comparison of multiple models aided interpretation of differences between the models, with these differences relating mostly to model characteristics that were evident in baseline model associations and baseline incidence in absence of vaccine. These characteristics include differences in relations between parasite prevalence and clinical incidence; case definitions; assumptions about rates of immune acquisition, immune decay, mechanisms of immunity; and differences due to datasets used for parameterisation.

Our analysis has some limitations. First, although the estimated vaccine efficacy and waning profile was similar across the models, these profiles diverged after 18 months of follow-up. The predicted long-term impact of the vaccine will inevitably depend on the pattern of these profiles and hence they should be updated with extended follow-up data. Second, whereas the models reproduce vaccine efficacy estimates from the trial, the projections of impact on disease and mortality are based on previous model-fitting to historical data relating clinical and severe incidence to mortality, in the assumption that associations in settings with more restricted access to health care are similar to those in settings with good access. Unlike the trials of insecticide-treated nets,^27^ which showed statistically significant reductions in malaria mortality, there was no significant impact on malaria mortality or all-cause mortality in the RTS,S trial. This outcome is likely to be due to the significantly lower levels of overall mortality in trial participants with high access to care than in those with low access. Nevertheless, the modelled estimates of RTS,S impact on mortality need further validation, and future studies done after implementation should include monitoring of impact on mortality. Third, we assumed that vaccination would be implemented at 6–9 months with three or four doses, yet actual implementation schedules and coverage might differ (including more than four doses). Over this age range, naturally acquired immunity develops rapidly and physiological effects can also affect the maturity of the vaccine-induced antibody response.^28^ Fourth, only two of the models allowed for possible indirect effects on transmission, with one model predicting indirect effects in settings with low malaria prevalence. Although the main aim of the vaccine is to provide direct protection, assessment of any indirect effects will be important in the post-licensure phase. Finally, we did not include productivity losses to households, and costs of immunisation and disease management were considered from a health-system perspective in our economic analysis. According to WHO guidelines,^29^ the societal perspective is generally preferred; however, in practice, this approach is rarely taken because not all costs are available. In general, addition of costs beyond the health system would increase the cost-effectiveness of vaccination, making our estimates lower. Despite these limitations, our results can help inform decisions about where to implement RTS,S to have the greatest impact.

WHO coordinated this model comparison process as an important contributory factor to the broader assessment processes related to this vaccine. These models did not incorporate safety aspects, as is standard for cost-effectiveness and impact models at the pre-licensure stage.^7^ These safety aspects include immediate reactogenicity including fever (noted with many vaccines), the identified risk of febrile convulsion within 7 days of vaccination (also not unique to this vaccine), and the potential risk of meningitis (which has not been causally related to vaccination), and are outlined in the June, 2014, report of the WHO Global Advisory Committee on Vaccine Safety.^30^ The WHO policy recommendation processes underway take all these elements into account as part of an overall assessment of benefits and harms, resource use and value for money, equity impacts, feasibility, and acceptability.

## Figures and Tables

**Table 1:** Assumed demographics, implementation coverage, and vaccine efficacy profiles

	**Harmonised comparison**
Demographics	Constant population size and demography based on the life table for Butajira, Ethiopia, with an average life expectancy at birth of 46·6 years.^15^
Transmission intensity	Parasite prevalence in 2–10 year olds between 3% and 65%, representing current transmission levels in Africa.^2^
Seasonality	Perennial transmission (no seasonality).
Case management	Effective coverage (ie, treatment with parasitological cure) for clinical malaria is 45%. Access to care for severe malaria varied by model.
Other interventions	Predictions assume that current interventions in place at the start of vaccination remain at static levels.
Vaccine efficacy	Model estimates of RTS,S efficacy against infection profiles based on fitting to phase 3 trial data (appendix pp 22–30).
Vaccine schedule	Three doses of vaccine given at 6, 7·5, and 9 months (6–9 month implementation) with a fourth dose at month 27 (6–9 month with fourth dose). The first two doses of the primary series are assumed to have 0% efficacy.
Vaccine coverage	90% coverage assumed for the three-dose schedule; we assumed a 20% drop-off in coverage for the fourth dose (72% coverage).
Cost of RTS,S vaccination	Vaccine and immunisation supplies including freight and wastage. The same costs were applied to all settings. These costs were estimated at US$6·52 per dose at vaccine price of $5, $2·69 per dose at vaccine price of $2, and $12·91 at vaccine price of $10 (appendix pp 33–34).
Cost of malaria case management	Costs are estimated by severity of illness and cover first-line antimalarial drugs, diagnostics, and related supplies including freight and wastage. We assumed full compliance and adherence with the age dosage. The same costs were applied to all settings, ranging from $1·07 to $2·27 per uncomplicated case, and from $21·78 to $55·58 per severe case (appendix pp 33–34).

**Table 2:** Predictions of public health impact and cost-effectiveness of RTS,S for the 6–9 month three-dose and four-dose immunisation schedules at 15 years of follow-up in regions with a parasite prevalence in 2–10 year olds of 10–65%

		**Three-dose schedule**	**Four-dose schedule**
Proportion of clinical cases averted in children younger than 5 years		16·2% (7·3–24·1)	21·1% (7·9–30·6)
Proportion of deaths averted in children younger than 5 years		13·8% (5·3–21·4)	18·0% (6·0–29·1)
Clinical cases averted per 100 000 fully vaccinated children		93 940 (20 490–126 540)	116 480 (31 450–160 410)
Deaths averted per 100 000 fully vaccinated children		394 (127–708)	484 (189–859)
Incremental benefit*			
	Clinical cases	..	22% (3 to 49)
	Deaths	..	31% (−1 to 53)
ICER per clinical case averted (in US$)			
	$2 per dose	$13 (7–88)	$10 (6–93)
	$5 per dose	$30 (18–211)	$25 (16–222)
	$10 per dose	$61 (31–415)	$51 (28–437)
ICER per DALY averted (in US$)			
	$2 per dose	$35 (16–112)	$38 (18–97)
	$5 per dose	$80 (44–279)	$87 (48–244)
	$10 per dose	$147 (90–556)	$154 (99–487)

Data are median (range) across the models' medians. ICER=incremental cost-effectiveness ratios.

* Proportion of additional events averted with four-dose versus three-dose immunisation schedule.
