# Supplementary material for: Public health impact and cost-effectiveness of the RTS,S/AS01 malaria vaccine: a systematic comparison of predictions from four mathematical models
Source: Lancet. 2016 Jan 23;387(10016):367–75. doi: 10.1016/S0140-6736(15)00725-4 (PMC4723722; doi:10.1016/S0140-6736(15)00725-4)
Supplement: Supplementary appendix [file mmc1.pdf]

THE LANCET

Supplementary appendix

This appendix formed part of the original submission and has been peer reviewed. We post it as supplied by the authors.

Supplement to: Penny MA, Verity R, Bever CA, et al. Public health impact and cost-effectiveness of the RTS,S/AS01 malaria vaccine: a systematic comparison of predictions from four mathematical models. *Lancet* 2015; published online Nov 5. http://dx.doi.org/10.1016/S0140-6736(15)00725-4.

The public health impact and cost-effectiveness of malaria vaccine candidate RTS,S/AS01: a systematic comparison of predictions from four mathematical models

Supplementary appendix

Melissa A. Penny[1,2]*§, Robert Verity[3]§, Caitlin A. Bever[4]§, Christophe Sauboin[5]§, Katya Galactionova[1,2], Stefan Flasche[6], Michael T. White[3], Edward A. Wenger[4], Nicolas Van de Velde[5], Peter Pemberton-Ross[1,2], Jamie T. Griffin[3], Thomas A. Smith[1,2], Philip A. Eckhoff[4], Farzana Muhib,[7] Mark Jit[6,8], Azra C. Ghani[3]

[1]Department of Epidemiology and Public Health, Swiss Tropical and Public Health Institute, 4051, Basel, Switzerland
[2]University of Basel, Petersplatz 1, Basel, Switzerland
[3]MRC Centre for Outbreak Analysis and Modelling, Imperial College London, London, UK
[4]Institute for Disease Modelling, Bellevue
[5]GSK Vaccines, Wavre, Belgium
[6]London School of Hygiene and Tropical Medicine, Department of Infectious Disease Epidemiology, Keppel Street, WC1E 7HT, London, UK
[7]PATH, 455 Massachusetts Ave NW, Suite 1000, Washington DC 20001
[8]Modelling and Economics Unit, Public Health England, 61 Colindale Avenue, London NW9 5EQ, UK
§ authors contributed equally

* Corresponding author:
Dr Melissa A. Penny
Swiss Tropical and Public Health Institute
Socinstrasse 57
4051 Basel, Switzerland
e-mail: melissa.penny@unibas.ch

TABLE OF CONTENTS

1 Model descriptions and baseline comparison of malaria models 6
1.1 Models and harmonization assumptions 6
1.1.1 Models of malaria epidemiology and vaccine impact 6
1.1.2 Demographics 6
1.1.3 Transmission settings and access to treatment 6
1.2 Relationships with prevalence 7
1.2.1 EIR-parasite prevalence relationships 7
1.2.2 Prevalence-incidence relationships 8
1.3 Age-incidence relationships 8
1.4 Epidemiological relationships to aid interpretation of impact of malaria interventions across the models 8
2 Supplementary methods and results: vaccine properties determined from Phase III clinical data 22
2.1 Summary estimates of the vaccine efficacy against infection and duration of response using Phase 3 clinical trial data 22
2.1.1 GSK Summary of methods and resulting vaccine properties 24
2.1.2 EMOD DTK Summary of methods and resulting vaccine properties 26
2.1.3 Imperial Summary of methods and resulting vaccine properties 27
2.1.4 OpenMalaria Summary of methods and resulting vaccine properties 30
3 Cost estimates for economic analysis of RTS,S 33
3.1 Program costs 33
3.2 Treatment health savings 34
3.3 Incremental cost-effectiveness ratios 34
4 Additional vaccine public health impact and cost-effectiveness results 37
4.1 DALY calculations 37
4.2 Additional Results 37
4.2.1 Vaccine public health impact 37
4.2.2 Cost effectiveness 38
5 Sensitivity analysis of RTS,S cost-effectiveness 51
5.1 Harmonization assumptions 51
5.2 Sensitivity analysis 51
6 References 54

LIST OF FIGURES

Figure S1.1 Relationship between EIR and PfPR2-10 in three models.. 10

Figure S1.2 Yearly incidence of clinical (uncomplicated) malaria as a function of *Pf*$PR_{2\text{-}10}$ displayed by model and age group. 10

Figure S1.3 Yearly incidence of total severe malaria as a function of *Pf*$PR_{2\text{-}10}$, displayed by model and age group. 11

Figure S1.4 Yearly incidence of total hospitalised malaria as a function of *Pf*$PR_{2\text{-}10}$, displayed by model and age group. 11

Figure S1.5 Yearly number of malaria-related deaths as a function of *Pf*$PR_{2\text{-}10}$, displayed by model and age group.. 12

Figure S1.6 Yearly incidence of clinical malaria as a function of age, displayed by transmission intensity (*Pf*$PR_{2\text{-}10}$) and model. ….12

Figure S1.7 Yearly incidence of total severe malaria as a function of age, displayed by transmission intensity (*Pf*$PR_{2\text{-}10}$) and model....13

Figure S1.8 Yearly incidence of total hospitalised malaria as a function of age, displayed by transmission intensity (*Pf*$PR_{2\text{-}10}$) and model. 13

Figure S1.9 Yearly incidence of malaria-related deaths as a function of age, displayed by transmission intensity (*Pf*$PR_{2\text{-}10}$) and model. 14

Figure S2.1 Predicted best fit RTS,S efficacy against infection profiles after the third dose of the primary course, as estimated from Phase III data in children receiving their first dose between 5 and 17 months....24

Figure S2.2 Predicted efficacy against clinical disease by trial site compared to Phase III reported for the 5-17 month cohort using best-fitted vaccine profile from GSK....25

Figure S2.3 Predicted efficacy against clinical disease by trial site compared to Phase III reported for the 5-17 month cohort with boost (4th dose) using best-fitted vaccine profile from GSK 25

Figure S2.4 Predicted efficacy against clinical disease by trial site compared to Phase III reported for the 5-17 month cohort using best-fitted vaccine profile from EMOD DTK....27

Figure S2.5 Predicted efficacy against clinical disease by trial site compared to Phase III reported for the 5-17 month cohort with boost (4th dose) using best-fitted vaccine profile from EMOD DTK 27

Figure S2.6 Predicted efficacy against clinical disease by trial site compared to Phase III reported for the 5-17 month cohort using best-fitted vaccine profile from Imperial....29

Figure S2.7 Predicted efficacy against clinical disease by trial site compared to Phase III reported for the 5-17 month cohort with boost (4th dose) using best-fitted vaccine profile from Imperial 30

Figure S2.8 Predicted efficacy against clinical disease by trial site compared to Phase III reported for the 5-17 month cohort using best-fitted vaccine profile from OpenMalaria 31

Figure S2.9 Predicted efficacy against clinical disease by trial site compared to Phase III reported for the 5-17 month cohort with boost (4th dose) using best-fitted vaccine profile from OpenMalaria. 32

Figure S4.1: Clinical cases averted per 100,000 fully vaccinated children stratified by age group and *Pf*$PR_{2\text{-}10}$. 48

Figure S4.2: Severe cases averted per 100,000 fully vaccinated children stratified by age group and *Pf*PR_{2-10}. 48

Figure S4.3: Hospitalised cases averted per 100,000 fully vaccinated children stratified by age group and *Pf*PR_{2-10}..49

Figure S4.4: Deaths averted per 100,000 fully vaccinated children stratified by age group and *Pf*PR_{2-10}..49

Figure S4.5: The cumulative number of either clinical cases, severe cases, hospitalised cases, death or DALYs averted per 100,000 fully vaccinated children within 15 years after the start of RTS,S vaccination in a 6 to 9 months schedule with a 4th dose...50

Figure S4.6: Cost effective planes comparing the net program costs of RTS,S with the DALYs averted. Showing total net incremental costs and DALYs averted for use of RTS,S within a 6 to 9months schedule over routine malaria control and incremental costs and DALYs averted of RTS,S with and without 4th dose are shown for different transmission settings and vaccine prices. 50

Figure S5.1: OpenMalaria one-way sensitivity and scenario analysis of RTS,S cost-effectiveness.............53

LIST OF TABLES

Table S1.1. Detailed descriptions of the models 15

Table S1.2 Malaria case definitions in the four models 19

Table S1.3 Summary of variability represented in predictions from each model group in baseline predictions 21

Table S2.1 Description of best-fit profile of vaccine efficacy against infection by model group from analysis of the Phase III data 23

Table S2.2: Imperial estimates of parameters describing the dynamics of RTS,S induced anti-CSP antibodies and the dose-response relationship. 29

Table S3.1: Prices, quantities, and unit costs for calculating cost of vaccine introduction 33

Table S3.2: Cost per dose administered by vaccine price. 34

Table S3.3: Prices, quantities, and unit costs for calculating cost of uncomplicated malaria episodes 34

Table S3.4: Prices, quantities, and unit costs for calculating cost of severe malaria episodes 35

Table S3.5: Cost of malaria case management by age group and severity of illness (USD, 2013). Malaria case management costs cover antimalarials and related medication and supplies, including freight and wastage. 36

Table S4.1: Deaths averted per 100,000 fully vaccinated and percentage of under 5 deaths averted by RTS,S for 6-9 month immunization schedule with or without 4th dose at 15 years follow-up for representative parasite prevalence settings (*Pf*PR_{2-10}). 39

Table S4.2: Clinical cases averted per 100,000 fully vaccinated and proportion of clinical cases averted by RTS,S for 6-9 month immunization schedule with or without 4th dose at 15 years follow-up for representative transmission settings (*Pf*PR_{2-10}). 40

Table S4.3: Cost per DALY averted by RTS,S for 6-9 month immunization schedule with or without 4th dose at 15 years follow-up for representative parasite prevalence settings (*Pf*PR_{2-10}). 41

Table S4.4: Deaths averted per 100,000 fully vaccinated and percentage of under 5 deaths averted by RTS,S for 6-9 month immunization schedule with or without 4th dose at 15 years follow-up for low prevalence representative transmission settings (*Pf*PR_{2-10}). 43

Table S4.5: Cost per DALY averted by RTS,S for 6-9 month immunization schedule with or without 4th dose at 15 years follow-up for low prevalence representative transmission settings (*Pf*PR_{2-10}). 44

Table S4.6: Summary predictions of public health impact and cost-effectiveness of RTS,S for 6-9 month immunization schedule with or without 4th dose at 15 years follow-up. 46

Table S5.1: List of parameters and ranges varied in one-way sensitivity analysis 51

Table S5.2: OpenMalaria one-way sensitivity of RTS,S cost-effectiveness as ICER per DALY averted by transmission level. 52

1 Model descriptions and baseline comparison of malaria models

Prior to carrying out model fitting and projecting vaccine impact, we compared the four mathematical models in the absence of any intervention other than treatment of malaria cases. The supplementary methods detail the key features of each of the models, with a focus on the assumptions, outputs and case definitions that lead to differences between model predictions. Details of the harmonization process are described, and finally baseline relations between key metrics such as parasite prevalence, entomological inoculation rate (EIR), clinical and severe incidence and mortality are presented.

1.1 Models and harmonization assumptions

1.1.1 Models of malaria epidemiology and vaccine impact

The models of *Plasmodium falciparum* malaria used in this study were developed independently by four modelling groups (Institute for Disease Modelling (IDM, model name EMOD DTK) [9], GlaxoSmithKline Vaccines (GSK) [16], Imperial College London (Imperial)[12] and Swiss Tropical and Public Health Institute (Swiss TPH, model name OpenMalaria[18])). Box S1.1 and Table S1.1 provide a description of each of the models and references for the technical details.

There are some differences in case definitions between the models as listed in Table S1.2 and further described in Box S1.2 in relation to disease definitions for *Plasmodium falciparum*.

The structural differences between the models mean that different sources of uncertainty are captured. These are listed in Table S1.3.

1.1.2 Demographics

Demographics of simulated human populations in the models were harmonised with birth cohort and population sizes based on common values. For Imperial the age structure of the population was derived from the life table for Tanzania 2010. For OpenMalaria a similar distribution was used, although with a higher mortality in the first year of life. For GSK and EMOD DTK a simple geometric distribution was used that provided a close match to other groups. In all models the total number of simulated individuals was 100,000 with a non-growing static population for baseline projections.

1.1.3 Transmission settings and access to treatment

We explored baseline parasite prevalence in 2-10 year olds ($Pf\mathrm{PR}_{2\text{-}10}$) ranging from 3% to 75%. Three of the models are driven by EIR (EMOD DTK, Imperial and OpenMalaria) and for those models the corresponding EIR ranged from 0 to 512 infectious bites per person per year. However, the specific relationship between EIR and $Pf\mathrm{PR}_{2\text{-}10}$ varied between models (see Figure S1.1). In all simulations a flat seasonal profile was used. All models were assumed to be in endemic equilibrium prior to introduction of the vaccine.

We assumed 45% of clinical episodes with patent malaria were successfully treated. In the Imperial model this was implemented as 52% access to ACT or non-ACT treatment (26% each), with efficacies of 95% and 75% respectively. For OpenMalaria 45% reflects access via both informal and formal care. Some aspects of the OpenMalaria model may be particularly sensitive to the access to care assumptions, and for this reason a sensitivity analysis of 15% access to effective care was carried out when characterising the relationship between EIR and $Pf\mathrm{PR}_{2\text{-}10.}$

Box S1.1: Descriptions of the four malaria models

GSK is a static and stochastic individual-based cohort model calibrated to reproduce the age-incidence of clinical malaria from the control arm of the RTS,S Phase 3 trial and age-distribution[1]. Health states of individuals in the cohort follow a Markov process with 5-day cycles and a time horizon of 5 years. Clinical malaria is predicted from parasite densities using MAP categories for low, moderate and high transmission[2]. Human infectiousness is not included. Vaccination is added as an additional immunity component which further reduces infection compared with current interventions (mainly bednet use and treatment). The three categories of transmission considered are (PfPR$_{2\text{-}10}$<5%, 5<=PfPR$_{2\text{-}10}$<=40%, PfPR$_{2\text{-}10}$>40%). Heterogeneity in exposure among individuals is included; for each transmission level, some individuals are almost never exposed while others have twice the mean exposure.
Institute for Disease Modelling – EMOD DTK is a discrete, stochastic, individual-based model of malaria in either local or spatially distributed settings. The vector module supports closed-loop cycles of mosquito development and blood-feeding for multiple independent species with temperature- and rainfall-dependent larval dynamics and sporogony[3]. The individual infection and immunity modules track the distribution of parasites by surface-antigen type with both innate and antigen-specific adapted immune responses, while human infectiousness is calculated directly from the mechanistic dynamics of parasite densities[4]. Blood-stage immunity is acquired through cumulative exposure to the set of unique and cross-reactive antigens in the parasite population[6] with heterogeneity in individual biting rates included. The model accounts for the combined effect of an extensive set of both vector- and human-directed interventions[7,8]. The relationships between transmission intensity, parasite prevalence, clinical episodes, and severe disease were calibrated to historical study-site data, and an ensemble of model parameterizations sampled from the posterior probability distribution[9]. As in that work, the present analysis uses a simplification of the vector module, which models transmission directly as a periodic function of force of infection. That is, in this framework the human infection reservoir does not modulate the transmission intensity.
Imperial College. The model is a stochastic, individual-based simulation of a single population of humans linked to a stochastic compartmental model for mosquitoes[10]. The human infection process tracks individuals through stages of infection, with pre-erythrocytic and blood-stage immunity incorporated to capture the changing patterns of severe disease, clinical diseases and asymptomatic infection with age and exposure. The vector model includes larval stages as well as adult female mosquitoes to capture the feedback of vector control that kills adults on the population dynamics[11]. Human infectiousness is related to asexual parasite dynamics and lagged to allow for development of gametocytes. Multiple vector species and heterogeneity in exposure is included. The model has been extensively fitted to data on the relationship between the entomological inoculation rate (EIR) and parasite prevalence, clinical disease, severe disease and deaths using Bayesian methods[12,13]. The model captures the combined effect of multiple interventions, including first-line treatment, LLINs and the RTS,S vaccine.
Swiss TPH – OpenMalaria is a stochastic, individual-based, single location simulation model of malaria in humans[14] linked to a deterministic models of malaria in mosquitoes[15]. The simulation model includes sub-models of infection of humans[17], blood-stage parasite densities[19], infectiousness to mosquitoes as a lagged function of asexual parasite density[20], incidence of morbidity including severe and hospitalisation[5,21] and mortality[5]. Pre-erythrocytic and blood-stage immunity comprise separate sub-models, with blood-stage immunity predominating as infection-blocking immunity occurs only in those with very high cumulative exposure[19]. An ensemble of 14 model variants with varying assumptions is available[18]. These models include different assumptions for decay of natural immunity, greater within-host variability between infection and entomological exposure, heterogeneity in transmission and heterogeneity in susceptibility to co-morbidities. In this work only six of the ensemble models were used and transmission is modelled through periodically varying vectorial capacity.

1.2 Relationships with prevalence

1.2.1 EIR-parasite prevalence relationships

The predicted relationship between EIR and PfPR$_{2\text{-}10}$ (Figure S1.1) differs slightly between the models, with variation consistent with the data used to inform them. For OpenMalaria and EMOD DTK the relationship is highly dependent on the level of case management, which has implications for the clinical disease incidence relationships. Higher treatment at a given EIR results in lower PfPR$_{2\text{-}10}$ and lower clinical disease[22]. This is due to the following: 1) parasite prevalence is indirectly related to malaria transmission because of effects of naturally acquired immunity and of heterogeneity in transmission rates and; 2) the

amount of treatment in the population truncates infections. If access to effective treatment is high, then prevalence may remain relatively low, even at high transmission levels and this also affects the relationships with incidence[22].

1.2.2 Prevalence-incidence relationships

Baseline relationships were established between *Pf*$PR_{2\text{-}10}$ and clinical incidence (Figure S1.2), severe incidence (total) (Figure S1.3), hospitalised cases (Figure S1.4), and mortality (Figure S1.5), each broken down into discrete age groups. For the OpenMalaria model extrapolations of the prevalence-incidence relationship are subject to a high degree of uncertainty at very high *Pf*$PR_{2\text{-}10}$ (>70%) and so results for these *Pf*$PR_{2\text{-}10}$ levels are not shown.

EMOD, Imperial and OpenMalaria all predict a decrease in incidence for ages greater than five years old at high *Pf*$PR_{2\text{-}10}$ (due to higher natural immunity from higher exposure earlier in life compared to low *Pf*$PR_{2\text{-}10}$ settings). This decrease is larger for OpenMalaria as incidence is higher overall owing to differences in case-definitions, model immunity assumptions, and the data used to parameterise the model. For hospitalised cases there is agreement between OpenMalaria and Imperial.

All models predict the majority of deaths to occur in the 0-5 year old age range at high transmission. At low transmission all models predict fewer deaths distributed over a broader age range. Differences between the models for mortality relationships can be attributed to the data used to parameterise them and the definition of a malaria death, as well as differences in model structure. In OpenMalaria deaths include those directly attributable to the disease and those caused by co-morbidities; in the other three models mortality represents direct malaria deaths (albeit potentially including a small fraction of deaths from other causes due to parameterisation against imprecise verbal autopsy data).

1.3 Age-incidence relationships

Acquisition of immunity and the speed with which this is acquired due to infection changes the observed incidence with age and transmission intensity[23]. In low *Pf*$PR_{2\text{-}10}$ incidence is seen in older ages compared to higher *Pf*$PR_{2\text{-}10}$ settings (Figures S1.7-S1.9). This is captured in all of the models, with incidence varying with age and transmission as a result of immunity acquisition and assumptions, with decreasing clinical incidence with age for higher transmission sites (Figure S1.6). The magnitude of clinical incidence differs across the models due to different case-definitions with two of the models (GSK and Imperial) modelling only one possible clinical case per infection. Total severe malaria by age and *Pf*$PR_{2\text{-}10}$ for three of the models is shown in Figure S1.7, hospitalised cases Figure S1.8, and malaria-related deaths in Figure S1.9, broken down by transmission intensity (defined in terms of *Pf*$PR_{2\text{-}10}$) and model. For the OpenMalaria model both direct deaths (deaths from malaria) and total deaths (including deaths from comorbidities associated with malaria) are shown.

1.4 Epidemiological relationships to aid interpretation of impact of malaria interventions across the models

The key epidemiological relationships presented in this model comparison aid in later interpretation of differences between model predictions of impact of malaria interventions such RTS,S. The age-incidence and prevalence-incidence relationships indicate the burden by age-group that it is possible to avert at a particular *Pf*$PR_{2\text{-}10}$ by an intervention. In particular, the difference in case definitions and the number of cases per infection between the models influences not just the burden but also the differences in predicted impact on clinical cases of any intervention at higher prevalence.

Box S1.2: Disease definitions for *P.falciparum* malaria

Infection with *Plasmodium falciparum* can lead to a number of different health outcomes, with a general trend towards decreasing pathology following the gradual acquisition of immunity by exposure and age. However, immunity is partial and hence episodes can occur across all age groups with asymptomatic carriage of parasite common in older children and adults. Young children are at risk of severe disease, the life-threatening form of malaria, which typically presents as either severe anaemia or cerebral malaria. There is no single clear definition of severe disease but in general this form of disease requires hospitalisation. Severe disease may onset very rapidly or can develop as a consequence of untreated clinical disease. Clinical disease (also referred to as mild or uncomplicated) is characterised by bouts of recurrent fever due to the cyclical burst of parasites during blood-stage infection. This is most often defined in research studies and trials on the basis of measured fever plus parasite density in the blood over some threshold, although again there is no single standard definition. Artemisinin combination therapies (ACTs) are indicated for treatment of clinical malaria, with hospitalisation only required in the case of additional complications. Asymptomatic infection is defined as the presence of parasites in the blood with no associated clinical symptoms. This can either be “detectable” (based on common diagnostics, including microscopy and rapid diagnostic tests) or “undetectable” (or sub-patent) which refers to low density infections detectable only using PCR methods.

Across the four models, the definition of **clinical malaria** is similar but the number of uncomplicated clinical cases that can occur per infection differs (for GSK and Imperial only one clinical case per infection can occur, whereas for EMOD DTK and OpenMalaria multiple episodes can occur). For **severe malaria**, three models (EMOD DTK, GSK, and OpenMalaria) provide predictions of total severe malaria cases, including those receiving in-patient treatment and those that would be classified as severe in the community. The incidence of **hospitalised malaria** cases is provided by all groups. In the EMOD DTK and OpenMalaria models, these are a subset of severe malaria cases; for GSK some hospitalised cases may also be clinical; for Imperial all symptomatic cases (both clinical and severe) are counted among the clinical, and hospitalised malaria is defined from studies that may include cases who do not meet a strict definition of severe malaria.

Mortality associated with malaria is predicted by all models, however there are differences by model, reflecting the wider uncertainty in the mortality attributable to malaria. The OpenMalaria model explicitly records total malaria deaths which includes those associated with co-morbidities, either inside or outside of hospital, and direct malaria deaths that are directly attributable to malaria. Indirect deaths are deaths that would be averted by eliminating malaria, but where malaria is not assigned as the main cause of death. These are mainly associated with co-morbidities, which would be expected to decrease with improved treatment of other diseases. In general predictions from OpenMalaria in this report are for all deaths. The Imperial model is fitted to data taken from verbal autopsy (VA). Thus, while this model does not include any explicit parameterisation of deaths due to co-morbidities, some indirect deaths are implicit in fitting to VA as VAs have issues of sensitivity and specificity. In the GSK model malaria mortality is estimated as a fixed percentage of severe cases (case-fatality risk), with values for hospitalised and non-hospitalised severe cases obtained from published literature[1]. Deaths with co-morbidities are included by GSK as these are included in the case definition of severe malaria. In the EMOD DTK analysis, malaria mortality was estimated as a fraction of severe cases, following the methodology outlined used previous by OpenMalaria[5]. For malaria deaths, given the uncertainty and variability in data used to parameterise the models, it is appropriate to consider the estimates across the models as a representation of uncertainty in predictions.

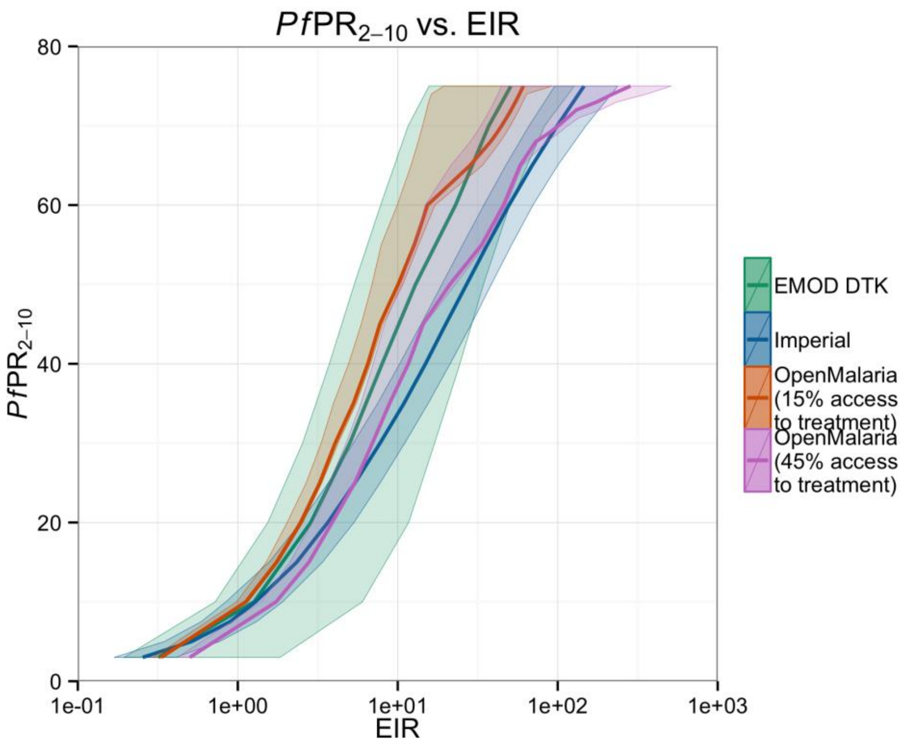

Figure S1.1 Relationship between EIR and PfPR2-10 in three models. Solid lines show medians and shaded regions show 95% credible intervals. EIR denotes the entomological inoculation rate.

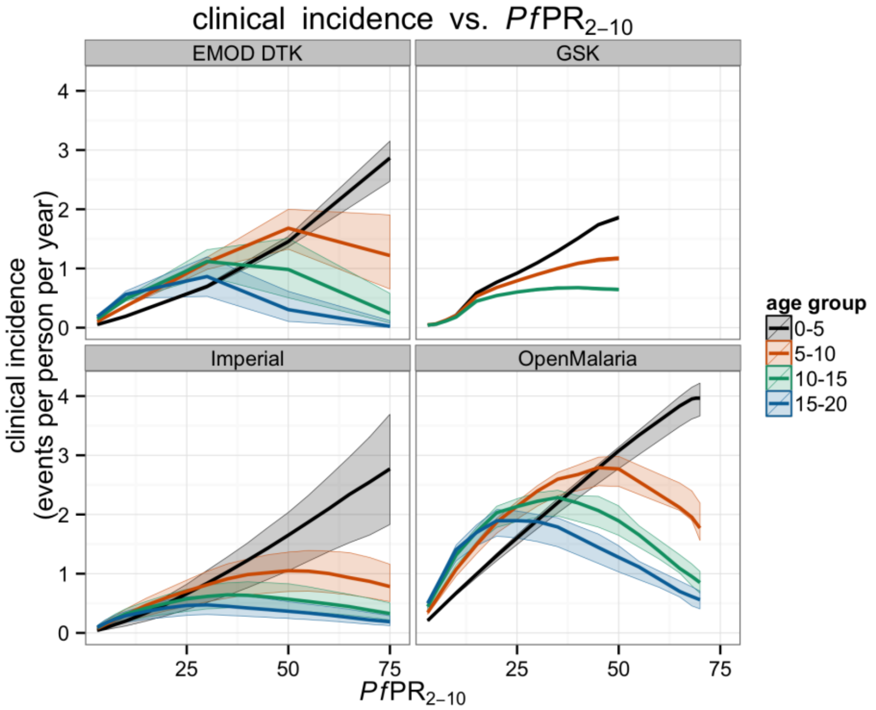

Figure S1.2 Yearly incidence of clinical (uncomplicated) malaria as a function of *Pf*PR$_{2\text{-}10}$ displayed by model and age group. Clinical incidence is presented in terms of the yearly number of events per person.

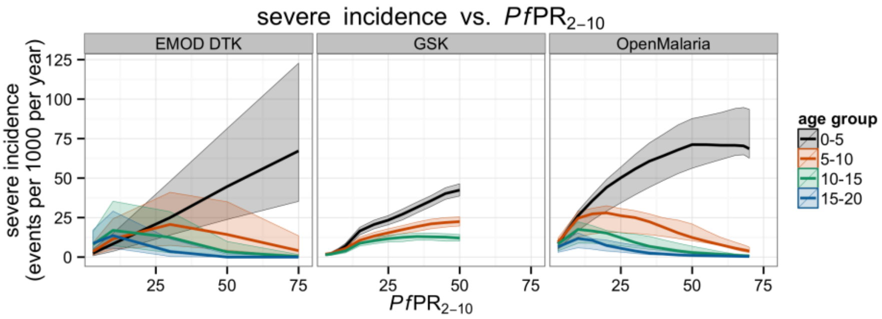

Figure S1.3 Yearly incidence of total severe malaria as a function of *Pf*PR$_{2\text{-}10}$, displayed by model and age group. Incidence is presented in terms of the yearly number of events in a population of 1000 individuals. (Imperial do not estimated severe cases)

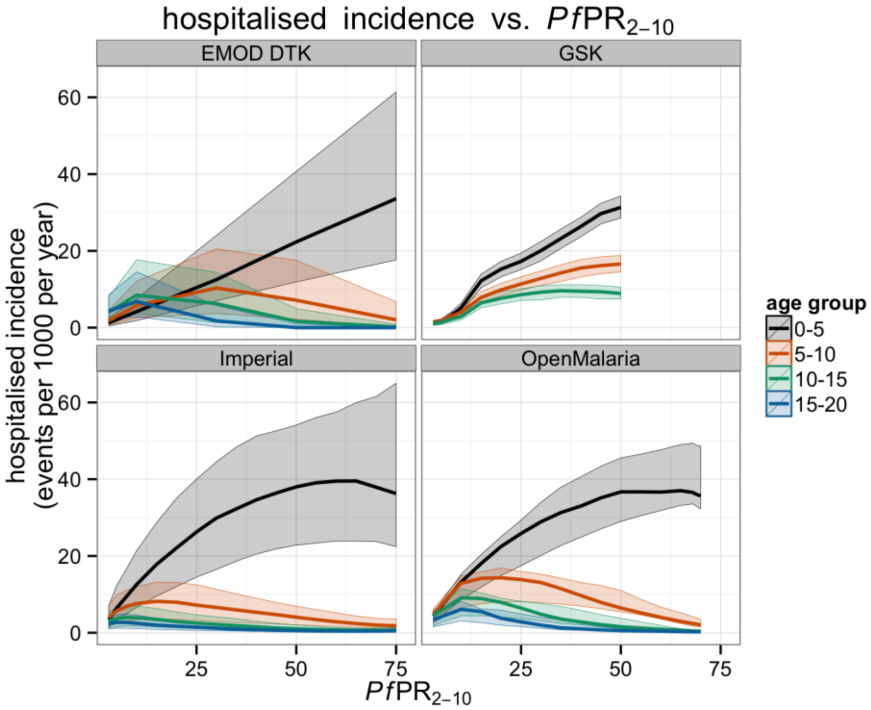

Figure S1.4 Yearly incidence of total hospitalised malaria as a function of *Pf*PR$_{2\text{-}10}$, displayed by model and age group. Incidence is presented in terms of the yearly number of events in a population of 1000 individuals.

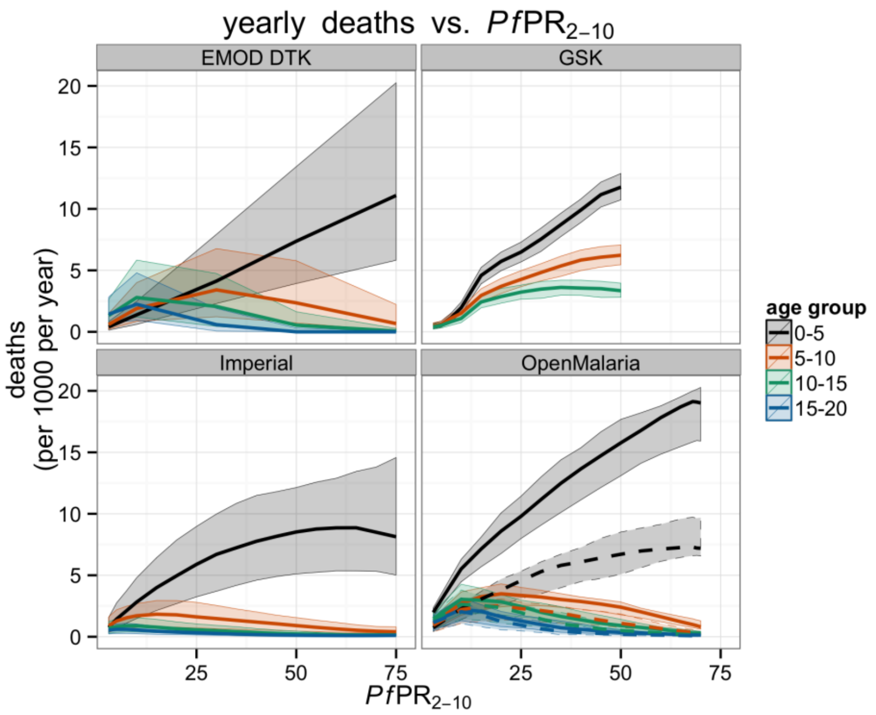

Figure S1.5 Yearly number of malaria-related deaths as a function of *Pf*PR$_{2\text{-}10}$, displayed by model and age group. Malaria mortality incidence is presented in terms of the yearly number of deaths in a population of 1000 individuals. For the OpenMalaria model both deaths directly attributed to malaria (dotted curve) and all deaths associated with malaria (including both deaths directly attributable to malaria and those associated with comorbidities) are shown (full line). See Box S1.2 for definitions of deaths attributable to malaria in the models.

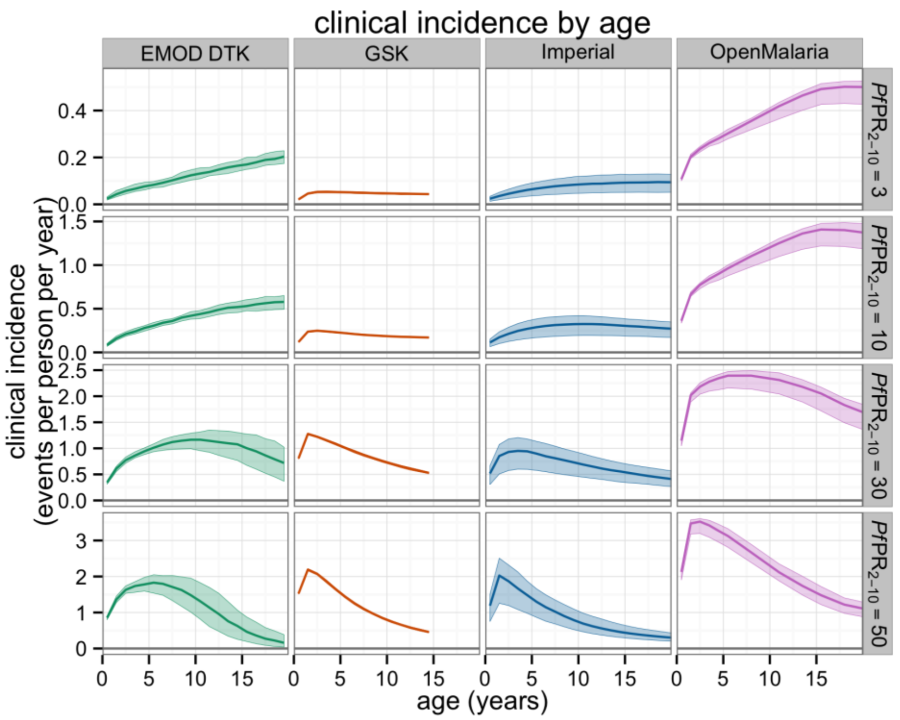

Figure S1.6 Yearly incidence of clinical malaria as a function of age, displayed by transmission intensity (*Pf*PR$_{2\text{-}10}$) and model. Clinical incidence is presented in terms of the yearly number of events per person.

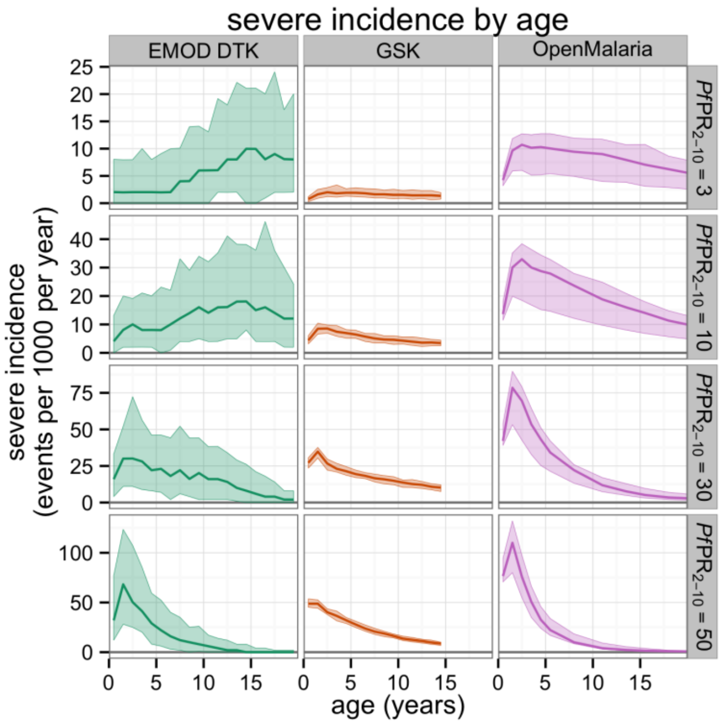

Figure S1.7 Yearly incidence of total severe malaria as a function of age, displayed by transmission intensity (*Pf*PR$_{2-10}$) and model. Incidence is presented in terms of the yearly number of events in a population of 1000 individuals. (Imperial only estimate hospitalised severe cases and so are not shown here).

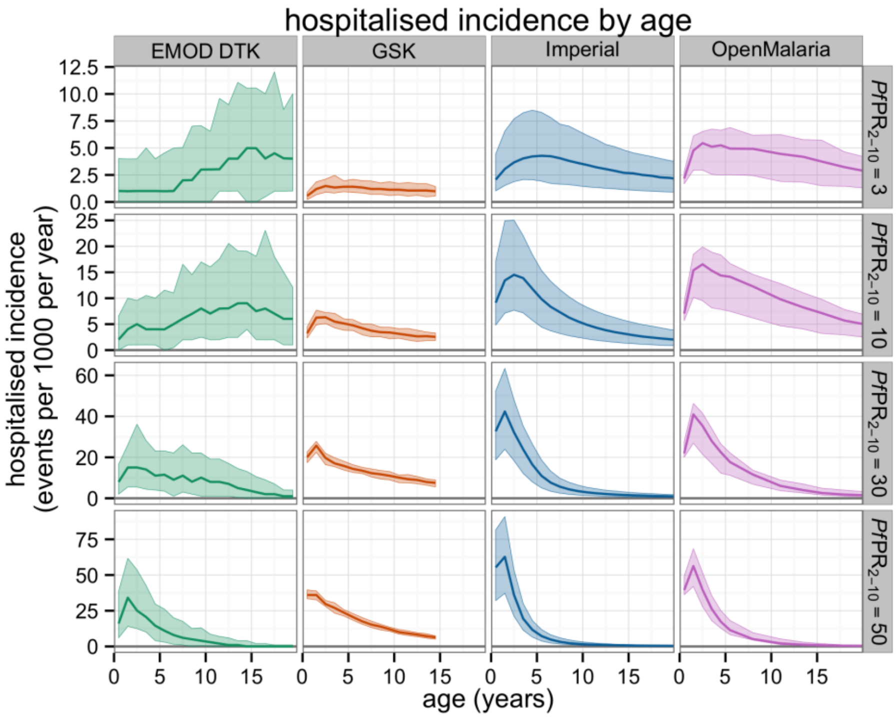

Figure S1.8 Yearly incidence of total hospitalised malaria as a function of age, displayed by transmission intensity (*Pf*PR$_{2-10}$) and model. Incidence is presented in terms of the yearly number of events in a population of 1000 individuals.

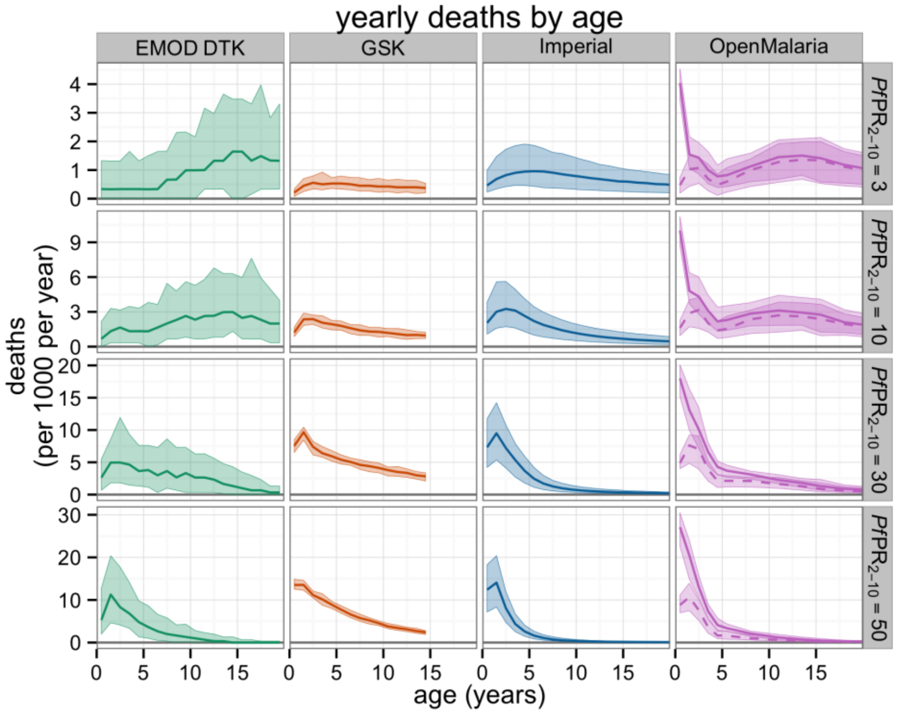

Figure S1.9 Yearly incidence of malaria-related deaths as a function of age, displayed by transmission intensity (*Pf*PR_{2-10}) and model. Malaria mortality incidence is presented in terms of the yearly number of deaths in a population of 1000 individuals. The dashed estimates for OpenMalaria represent direct malaria deaths, and the solid all malaria deaths (including those attributable to co-morbidities).

Table S1.1. Detailed descriptions of the models

	GSK	Imperial College	Institute for Disease Modelling – EMOD DTK	Swiss TPH – OpenMalaria
**Key representative publications**	Conference presentations[16,24]	Journal articles[10,12,13,25]	Journal articles[3,4,6-9 26]	Journal articles[14,17,21 5,15,18-20,27]
**Accessibility**	A deterministic version of the model has been developed in MS Excel which can be shared upon request	Interface to published model allows the user to run the model on their PC. Incorporates estimated prevalence, vector species, seasonality, ITN coverage and treatment at ADMIN1 level that the user can change. Current version released as part of the Elimination Scenario Planning Tool (WHO GMP) in April 2014. https://www1.imperial.ac.uk/malaria modelling/toolsdata/	Latest Windows release of EMOD malaria model and documentation can be downloaded from idmod.org/software	Code is open source. Runs on Windows, Linux and Mac. Full documentation is found at https://github.com/SwissTPH/open malaria/wiki<br>PATH Malaria Vaccine Initiative has a number of pre-populated simulations of one of the OpenMalaria models with a simple interface and supply, demand, cost information[28]
**Seasonality**	No	Yes	Yes	Yes
**Heterogeneity in exposure**	Yes	Yes	Yes	Yes
**Blood-stage parasite densities modelled**	No	No	Yes	Yes
**Parameterization for clinical incidence**	Fitted to RTS,S Phase III trial	Fitted to cross-sectional age-incidence data from 23 sites in Africa capturing differences between active and passive case detection [12]	Calibrations to 4 sites for parasitaemia (Nigeria, Tanzania), Dielmo and Ndiop (Senegal) [29] for age-incidence of clinical malaria	Calibration for age-incidence of clinical malaria for Dielmo and Ndiop in Senegal[29], and Idete, Tanzania[30]
**Parameterization for severe disease and mortality incidence**	Fitted to Phase III trial for severity and overall mortality using case fatality rate (CFR) from WHO report[31]	Severe disease model[13] fitted to data from northern Tanzania[32] and to severe disease vs. prevalence relationship from data of multiple sites[33]. Mortality due to malaria is based on Africa-wide data from verbal autopsy and parasite prevalence[34].	Age incidence of severe malaria fitted to 5 sites in The Gambia and Kenya[35]. Proportion of severe disease from anemia and cerebral malaria[36] CFR was normalized to match WHO death estimates.	Severe disease and mortality model[5] fitted to all-cause and cause-specific age-specific mortality from pre-LLIN and pre-ACT era, to hospitalisation rates by prevalence for multiple sites[33] and to age incidence of hospitalized severe malaria[35] (with age-specific CFR based on Tanzanian data[32])
**Vaccines interventions**	Pre-erythrocytic vaccines	Transmission blocking, pre-erythrocytic and combinations	Transmission blocking vaccines, blood stage vaccines, pre-erythrocytic and combinations	Transmission blocking vaccines, blood stage vaccines, pre-erythrocytic and combinations
**Pre-erythrocytic vaccine effect in the model**	Proportionate reduction in force of infection, exponential and bi-phasic	Proportionate reduction in force of infection. Vaccine efficacy decays	Proportionate reduction in force of infection, exponential decay.	Proportionate reduction in force of infection assuming beta distributed

	GSK	Imperial College	Institute for Disease Modelling – EMOD DTK	Swiss TPH – OpenMalaria
	decay	using exponential or biphasic decay, or using an antibody-based function informed by Phase II/III studies.		variation in efficacy. Various different assumptions concerning vaccine efficacy and its decay have been modeled, including exponential and biphasic-like (implemented via Weibull decay function)
**Vector control Interventions**	Assumes that parasite prevalence levels represents prevalence under current levels vector control interventions without modelling them explicitly	LLIN, IRS, Larval control (larviciding & pupaciding)<br>Novel interventions - GM mosquitoes, Ivermectin, Attractive Toxic Sugar Baits	LLIN, IRS, Larviciding-- effect depends on vector species-specific feeding behaviors Novel vector-control interventions: ivermectin, GM mosquitoes, individual and spatial repellents, oviposition traps, sugar-baited traps, etc.	LLIN, IRS, Larviciding, repellents and screening, zooprophylaxis, odour-baited traps, sugar-baited traps -- effect depends on vector species-specific feeding behaviors. Model includes loss of insecticide and development of holes in LLINs.
**Treatment interventions**	Treatment of clinical disease and severe disease.	Treatment of clinical disease and severe disease, by specified drug and diagnostic.<br>Mass screen and treat, IPTi, IPTc/SMC and IPTp/IST for separate pregnancy model	Drugs (routine access, mass administration, age- and risk-group targeting, diagnostic-guided administration)	Treatment of clinical disease and severe disease, by specified drug, facility level and diagnostic.<br>Mass screen and treat, MDA, IPTi, IPTc/SMC. Model allows for drug resistance.
**Spatial dynamic model**	No	Capacity to run full spatial model, including spatial interactions, although for this exercise model was run independently at ADMIN1 level and aggregated, thereby capturing spatial heterogeneity but not spatial interactions.	Yes	No
**Predictions for country or geographic area**	Yes	Yes (see above)	Yes	Yes. Based on MAP[37] prevalence, population and access to effective treatment by geographic area[38]
**Super-infections**	No	New infection takes priority over existing infection	Superinfections with each infection having its own antigenic repertoire (possibly partially overlapping)	Superinfections occur with summed parasite densities
	GSK	Imperial College	Institute for Disease Modelling – EMOD DTK	Swiss TPH – OpenMalaria
**Exposure**	Age dependent exposure, restricted to 0-10y. Susceptibility to infection increases with age	Exposure varies both by age and between individuals.	Configurable age-dependent exposure functions.	Age dependent exposure with non-linear function describing relationship between exposure and infection, with exposure rate varying

	GSK	Imperial College	Institute for Disease Modelling – EMOD DTK	Swiss TPH – OpenMalaria
				stochastically dependent on body surface area[17] .
**Infection-blocking immunity**	Infection-blocking immunity and immunity against severe malaria develop with the number of previous infections	Infection blocking immunity develops with exposure and age.	Minimal natural pre-erythrocytic immunity is attained through sustained exposure to infectious bites.	Infection-blocking immunity occurs only in those with very high cumulative exposure[17]
**Blood stage infections and immunity**	Blood stage immunity is acquired through exposure to blood stage infections, increasing with number of infections. Immunity acts against clinical and severe disease, with immunity to severe acquired faster than clinical disease.	Blood stage immunity develops with exposure and age, reducing both detectability of infection and onwards infectiousness.	Blood-stage immunity is acquired through the cumulative exposure to different malaria infections with varying but partly overlapping antigenic repertoires	Blood stage immunity develops with cumulative exposure to parasite densities and malaria infections. Effect of blood-stage immunity is to reduce parasite density. Conditional distributions of parasite densities are log-normal[19]
**Duration of infection**	Fixed duration of infection	Duration of infection is "Erlang-like" distribution (convolution of exponential distributions)	Duration of infection driven by strength of hyper-immune response to discrete repertoire of antigens presented by each clonal infection..	Duration of infection is log normal
**Clinical disease and history of exposure**	Immunity against clinical disease increases based on the number of previous infections (calibrated)<br>3 levels of risks of infection considered based on parasite prevalence [39]	A proportion of infected individuals go on to develop clinical disease. Immunity to clinical disease develops with exposure and age, and also has a maternally acquired component.	Clinical disease is triggered by pro-inflammatory cytokines in response to parasite density passing through a configurable pyrogenic threshold, down-regulated to specific antibody production.	Clinical diseases is triggered by parasite densities and individual pyrogenic threshold. Pyrogenic threshold is dependent on history of exposure, with cumulative exposure characterised by diversity of previous infections and cumulative parasite density[21].
**Decay of natural immunity**	No decay of naturally acquired immunity (calibrated)	Exponential decay of naturally acquired immunity	Capacity for antibody production to specific parasite antigens decays to memory levels upon clearing an infection	Original model included no decay of natural immunity. Three model variants in the ensemble include different functional forms for decay of immunity[18].
**Infectiousness and gametocyte models**	Human infectiousness not included, hence no change in transmission following intervention	Human infectiousness to mosquitos is a weighted sum over the different human infectious states. A time lag between asexual parasitemia and infectious gametocytemia accounts for the lag in gametocyte development.	Probability of infecting mosquito is sigmoidal function of gametocyte density. Inflammatory immune response limits infectiousness of individuals	Infectiousness depends on lagged asexual parasite densities and on presence of gametocytes in blood meal which is a stochastic function of gametocyte density. Both male and female gametocytes must be present to infect mosquito[20].
**Entomological models**	No	Vector control interventions include significant additional feedback of	Separate mosquito populations for each species. Mosquito feeding	Entomological model includes different species (or types) of

	GSK	Imperial College	Institute for Disease Modelling – EMOD DTK	Swiss TPH – OpenMalaria
		killing of adult mosquitoes on mosquito dynamics.	behavior (e.g. fraction of attempted indoor feeds) and larval-habitat preference are currently constant parameters for each species.	mosquitoes with explicit infection stages and heterogeneous survival probabilities depending on host, intervention and mosquito. Vector control interventions include feedback of killing of adult mosquitoes on mosquito population dynamics[15,40]. Effects of resistance to vector control on mosquito survival and infection included in the entomological model
**Outcomes from the models relevant for vaccine impact and cost-effectiveness**				
**Transmission related inputs and outcomes**	Input: Prevalence	Input: EIR<br>Output: Prevalence of infection by microscopy and PCR by specified age group	Input: EIR<br>Output: Prevalence of infection by specified age group	Input: EIR<br>Output: Prevalence of infection by microscopy and PCR specified age group
**Outcomes: Disease related (see Table S1.2 for case definitions)**	◆Clinical cases<br>◆All severe cases and hospitalized<br>◆Deaths due to malara<br>◆DALY<br>◆Cost-effectiveness	◆Clinical cases<br>◆Hospitalisation due to malaria<br>◆Deaths due to malara<br>◆DALYs<br>◆Cost-effectiveness	◆Clinical cases<br>◆Severe and hospitalized cases<br>◆DALYs (based on severe disease calibration above, with harmonization around total death numbers)<br>◆Cost-effectiveness	◆Clinical cases<br>◆All severe cases and hospitalized<br>◆Deaths (both directly attributable to malaria and indirect associated with co-morbidities)<br>◆DALYs (based on direct deaths or all deaths)<br>◆Cost-effectiveness

Table S1.2 Malaria case definitions in the four models

Malaria case definitions	GSK	Imperial	EMOD DTK	OpenMalaria
**Clinical uncomplicated malaria**	Based on secondary case definition 1 of the Phase 3 trial: children presenting with fever and parasite density >0/µL	The definition of uncomplicated malaria in the model is governed by the data that the model was fitted to. The model is calibrated to the incidence that is detected via daily active case detection. Case definitions in the input data fell into two groups: malaria symptoms plus any parasitaemia, or malaria symptoms plus a parasite density above a non-zero threshold. In the model, only one episode of clinical disease can occur with each new infection.	The definition of a clinical malarial incident is configurable in the EMOD model; for this study, a clinical incident begins when an individual's body temperature is raised by 1.5°C. Parasite density alone does not trigger a clinical incident, though temperature and parasite density are implicitly linked through the innate immune response. The clinical incident continues until the fever remains below 0.5°C for two weeks; this refractory period prevents the multiple recrudescent fever events typical to malaria from being recorded as multiple independent clinical incidents. [9]	An episode of uncomplicated malaria is a period during which an individual has symptoms caused by malaria parasites present at the time of illness, where the symptoms do not qualify as severe malaria. Uncomplicated cases are triggered by parasite densities over a pyrogenic threshold that is immunity dependent. The maximum length of the period of an episode is generally set to 30 days in the model and illness recurring within this period counts as the same episode. Illness recurring over a longer duration than this is counted as more than one episode. An illness caused by a pathogen other than malaria does not count as a malaria episode even if there is incidental parasitemia.
**Severe**	Based on secondary definition 1 from Phase 3 trial: parasite density of >5,000/µL and with one or more marker of severity (not excluding the presence of co-morbidities)	The age patterns of severe malaria incidence were fitted using data from Reyburn [32] and Marsh[33]. The overall incidence is calibrated to the study of Marsh and Snow, where the definition is hospitalised cases with malaria "parasitaemia and no other detectable cause for the clinical presentation".	An individual's current state is mapped onto three probabilities of diagnosis of severe malaria due to three underlying causes. An individual's current RBC count sets a probability of diagnosis due to anaemia and associated presentations, their current body temperature acts as a proxy for presentation of severe cerebral malaria, and their current parasitaemia level acts as a catch-all proxy for other complications (e.g., respiratory involvement). Similar protections to those described above regarding clinical incidents are in place to prevent a	Severe malaria is a potentially life-threatening disease, diagnosable by clinical or laboratory evidence of vital organ dysfunction, requiring in-patient care[41]. An episode of severe malaria is a period during which an individual has symptoms, qualifying as severe malaria, caused by malaria parasites present at the time of illness. As with uncomplicated malaria, the maximum duration of an episode is set to 30 days: illness recurring over a longer duration than this is counted as more than one episode.

			single severe presentation from being recorded as multiple incidents. [9]	
**Hospitalised malaria**	Malaria hospitalizations case definition 1 from Phase 3 trial: A medical hospitalization with confirmed parasite density >5,000/µL	Hospitalised malaria is the only severe malaria output (see above).	A hospitalized case refers to an individual with severe malaria that has been admitted for in-patient treatment.	Malaria hospitalisations are severe malaria episodes simulated as receiving in-patient care.
**Malaria mortality**	Malaria deaths are counted as a proportion of hospitalized severe malaria cases and community severe malaria cases	A proportion of severe cases are assumed to result in mortality in hospital, and a further scaling factor is used to extrapolate this result to total (inside or outside of hospital) deaths.	The EMOD model counts deaths that result directly from severe cases of malaria and in its current configuration does not account for mortality resulting from uncomplicated cases and/or comorbidity. Deaths in all settings (both hospital and community) are included.	Direct malaria deaths are severe malaria episodes that result in death.<br>Indirect malaria deaths are deaths that occur because of malaria infection but that do not satisfy the definition of direct malaria deaths. These comprise neonatal deaths secondary to malaria in pregnancy, and deaths resulting from interactions between pathogens where malaria plays an essential role, but the terminal illness does not satisfy the definition of severe malaria.

Table S1.3 Summary of variability represented in predictions from each model group in baseline predictions

Outputs	GSK	Imperial	EMOD DTK	OpenMalaria
**Baseline predictions including vaccine impact by transmission levels**	Stochastic uncertainty<br>Uncertainty in immunity acquisition<br>Heterogeneity of exposure	Minimal stochastic variability.<br>Uncertainty in immunity acquisition parameters (values drawn from posterior distribution given previous model fits).	Stochastic uncertainty<br>100 draws samples from a 12-dimensional parameter space spanning high-likelihood fits to clinical and severe malaria incidence, as described in McCarthy[9].<br>Uncertainty due to access to treatment is currently only demonstrated in the EIR-to-prevalence relationship.	Stochastic uncertainty<br>Model structural uncertainty limited to 6 models from model ensemble[18] (Uncertainty due to access to treatment not currently shown in vaccine impact predictions, however shown in the EIR to prevalence relationship)
**Vaccine impact for best-fit vaccine efficacy profiles in representative transmission levels**	Same as for baseline<br>Vaccine efficacy uncertainty	Same sources of variability as for baseline, plus uncertainty in vaccine-specific parameters (values drawn from posterior distribution given fits to Phase III trial data).	The above sources plus heterogeneity of exposure	Same sources or variability as for baseline.

2 Supplementary methods and results: vaccine properties determined from Phase III clinical data

Each of the models in this study was fitted independently to data from the long-term (32+ months) follow up of the Phase III clinical trial of RTS,S/AS01 in 11 sites. Both OpenMalaria and EMOD DTK used pooled 3-monthly incidence data from the intention-to-treat (ITT) population under the primary case definition. GSK used pooled 3-monthly incidence data from the ITT population under the secondary case definition. Imperial used individual-level data on incidence and antibody titres from the according-to-protocol (ATP) population under the primary case definition. The methodologies used to fit each of the models, along with model fits to each of the 11 trial sites, are detailed below.

2.1 Summary estimates of the vaccine efficacy against infection and duration of response using Phase 3 clinical trial data

Estimates of the initial efficacy against infection of the RTS,S/AS01 vaccine and its waning over time are shown by model in Table S2.1 and Figure S2.1. Despite differences in fitting approaches and transmission models used to parameterise the RTS,S efficacy profile, all four groups estimate a high initial post 3rd dose efficacy against infection (>75% Table S2.1) in the 5-17 month cohort. The estimated waning of efficacy during the first 12 months post 3rd dose is similar for all 4 groups. However, there is a divergence in the waning profile past one year after the 3rd dose: EMOD DTK and OpenMalaria suggest a more rapid decay than Imperial and GSK predictions.

For the 4th dose both Imperial and EMOD DTK estimate a higher initial efficacy and a slower waning of the vaccine than OpenMalaria and GSK. Both EMOD DTK and GSK assumed single exponential profiles for decay of the 4th dose efficacy, with EMOD DTK fitting a high initial response and faster decay whilst GSK fit a lower initial response and slower decay. OpenMalaria a priori assumed the waning profile (decay shape and rate of decay) for the 4th dose is the same as that following the initial 3 doses, hence their estimates are heavily weighted by data from the initial 18 months of follow-up in which there are more observations. Imperial estimate the decay to be slower than the initial decay as a consequence of a different ratio of the short-lived and long-lived components of the antibody responses. While the EMOD DTK model does not provide a mechanistic reason for slower decay, they also estimate a slightly longer half-life for the waning of the 4th dose.

There are two potential reasons for divergence in the waning profile. Firstly, the cohort of children enrolled in the study arms that received their first RTS,S dose between 5 and 17 months of age is split into the booster (4-dose) and no booster (3-dose) arms 18 months post dose 3, resulting in a 50% decrease in statistical power. The follow-up also extends only to 32 months (although the last category includes follow-up in some individuals up to 48 months). There is less power to estimate the second phase of the waning profile. Secondly, the groups made different parametric assumptions for the waning profiles (Table S2.1).

It is important to note that a comparison of estimated initial efficacy and waning profiles should be considered in conjunction with the corresponding model-dependent translation from efficacy against infection to efficacy against clinical disease. Figures (S2.2-S2.9) show the predicted efficacy against clinical disease from each model in each of the 11 trial sites using their respective best estimates of the efficacy against infection profile. For all models, the predicted efficacy against clinical malaria falls within the confidence intervals of the trial data.

Table S2.1 Description of best-fit profile of vaccine efficacy against infection by model group from analysis of the Phase III data

	5-17 month cohort initial efficacy against infection (at third dose)	5-17 month cohort decay of efficacy against infection	5-17 month cohort with 4th dose initial efficacy against infection at 4th dose	5-17 month cohort decay of efficacy against infection for 4th dose
**GSK**	83.5% (95% CrI: 57.9-91.2)	Bi-phasic exponential decay<br>1st phase: 10.3 months half life<br>2nd phase: 16.3 years half-life<br>Switch between phase occurs at 1.2y after dose 3	53.1%	Exponential decay with half-life of 3.7y
**EMOD DTK**	80%	13.5 months (single exponential)	40%	15 months (single exponential)
**Imperial**	75.2% (95% CrI: 71.0%, 78.9%)	Determined by decay of antibody titres as follows:<br><br>Half-life of short-lived antibodies: 45 (95% CrI: 42, 48) days<br>Half-life of long-lived antibodies: 591 (95% CrI: 557, 632) days<br>Proportion of short-lived antibodies 0.88 (95% CrI: 0.87, 0.89)	67.5% (95% CrI: 63.9%, 71.2%)	Determined by decay of antibody titres as follows:<br><br>Half-life of short-lived antibodies: 44 (95% CrI: 42, 48) days<br>Half-life of long-lived antibodies: 591 (95% CrI: 557, 632) days<br>Proportion of short-lived antibodies 0.70 (95% CrI: 0.68, 0.72)
**OpenMalaria**	91.1% (95% CrI. 74.5-99.7%)	Estimated half-life 7.32 months (95% CrI. 6-9.7 months)<br>Decay shape bi-phasic-like, described by Weibull decay shape parameter 0.69 (95% CrI 0.54-0.9)	49% (95% CrI 32-68.6%)	As per first 3 doses

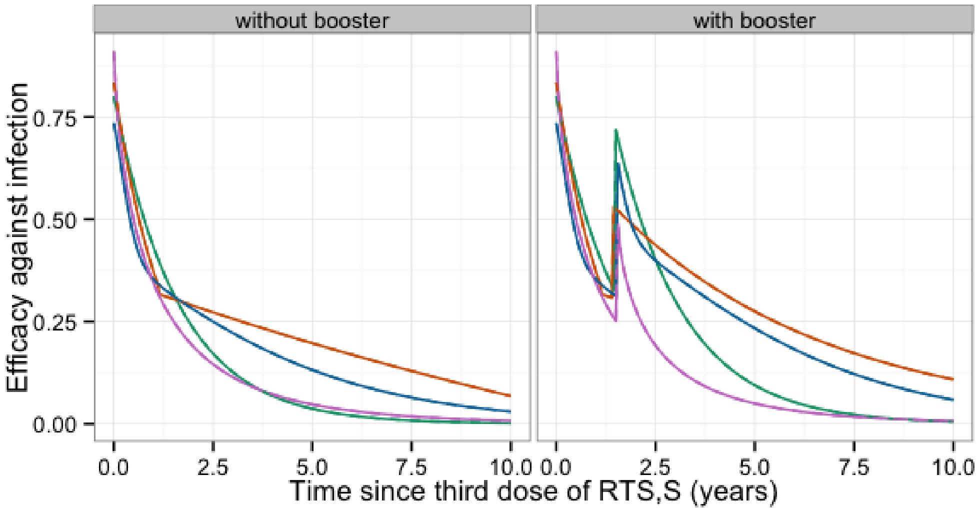

Figure S2.1 Predicted best fit RTS,S efficacy against infection profiles after the third dose of the primary course, as estimated from Phase III data in children receiving their first dose between 5 and 17 months.

Colours indicate groups (green EMOD DTK, orange GSK, blue Imperial and purple OpenMalaria) and panels the use of an 4th dose at 27 months of age (left panel without 4th dose, right panel with a 4th dose). Note that efficacy against infection translates differently into clinical efficacy for the four models (compare Figures S2.2 to S2.9 for the respective model fit to clinical disease data).

2.1.1 GSK Summary of methods and resulting vaccine properties

RTS,S efficacy profile (defined by the initial efficacy against infection and decay over time) was estimated using simultaneously (1) clinical efficacies by 3-monthly follow-up periods in the intention-to-treat (ITT) cohort based on the secondary case definition (parasite density of >0 and presence of fever) and (2) efficacies against severe malaria based on secondary case definition (presence of a marker of malaria severity and including co-morbidities) over the follow-up periods 3-8M, 9-14M, 15-20M, 21-32M and 33M-SE. Efficacies per clinical site were pooled into 3 transmission categories using definitions consistent with the Malaria Atlas Project (MAP). Low intensity is defined as parasite prevalence ($PfPR_{2\text{-}10}$) ≤5%, moderate intensity as $PfPR_{2\text{-}10}$5–40%, and high intensity as $PfPR_{2\text{-}10}$>40%. This definition is also consistent with the country-level distribution of children into each transmission category provided by MAP.

The RTS,S vaccine efficacy profile was determined by least-square procedure based on the minimization of a distance function summing squares of errors and weighing each data point by the corresponding 95% confidence interval widths.

Two different decay shapes were tested: single exponential or bi-phasic exponential. The half-life parameter of each exponential was estimated simultaneously with efficacy. For the bi-phasic decay, the time point for switching between exponentials is the fourth parameter estimated. No additional efficacy was considered against severe malaria or mortality. Bi-phasic decay was used in the final model as it better fitted trial data.

For the 4th dose efficacy and decay, clinical efficacies of 3-monthly periods of the ITT cohort were used. A single exponential decay was assumed. The same least square procedure was applied to determine the additional efficacy against infection at the time of 4th dose and half-life.

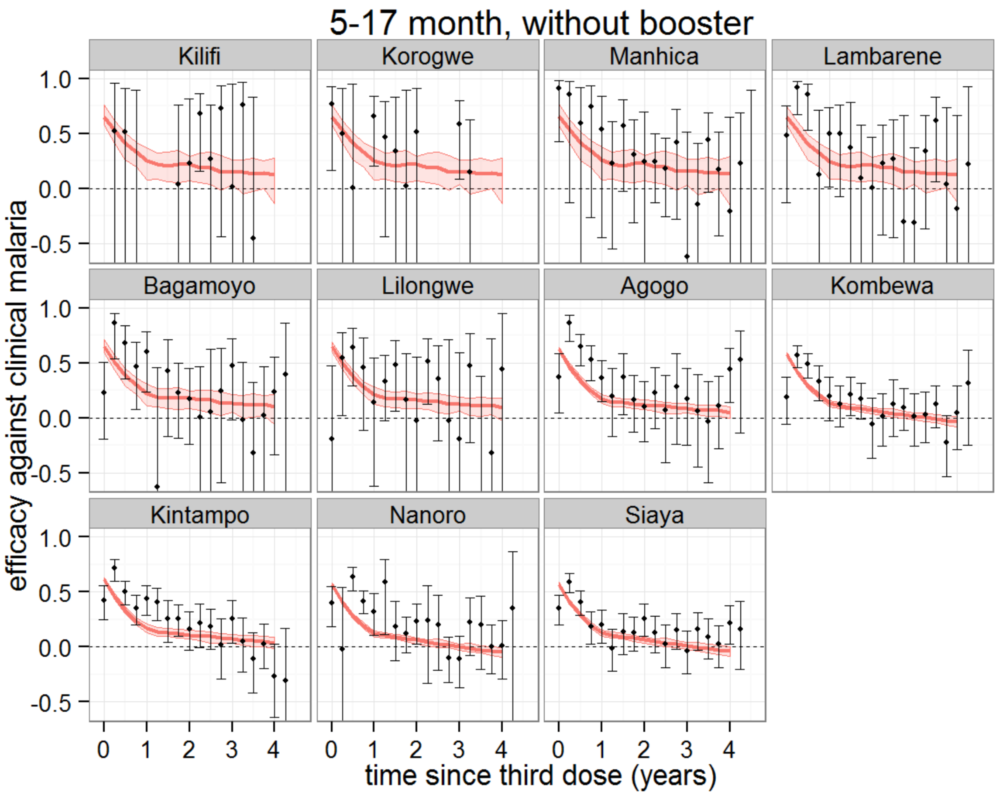

Figure S2.2 Predicted efficacy against clinical disease by trial site compared to Phase III reported for the 5-17 month cohort using best-fitted vaccine profile from GSK

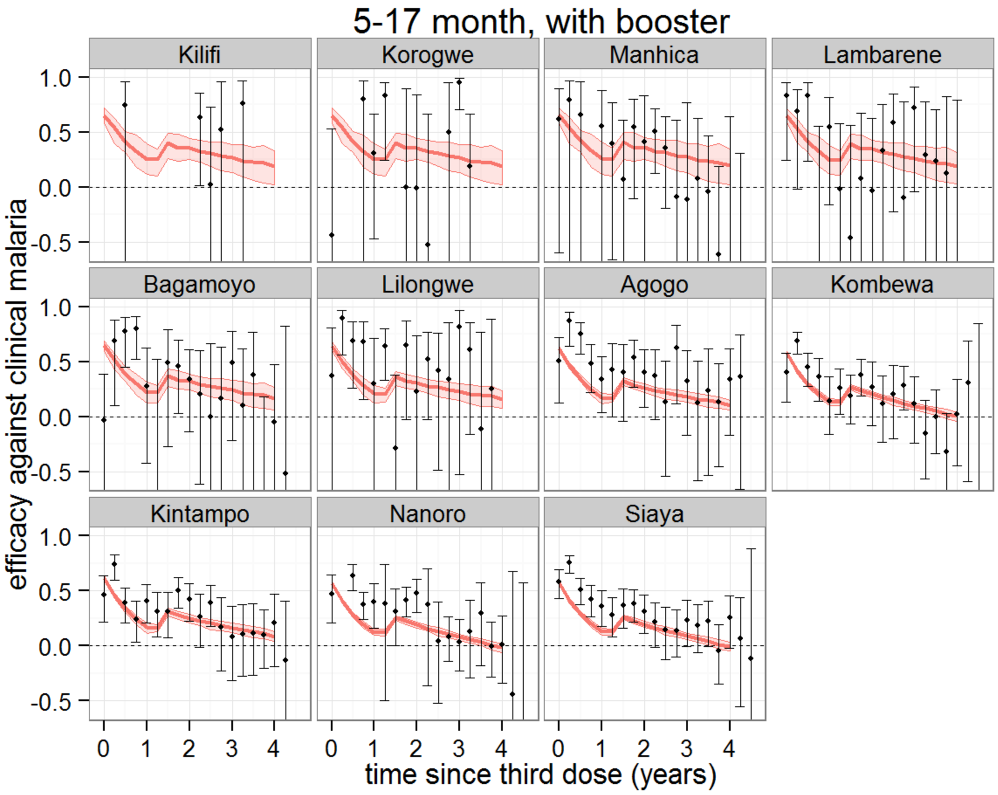

Figure S2.3 Predicted efficacy against clinical disease by trial site compared to Phase III reported for the 5-17 month cohort with boost (4th dose) using best-fitted vaccine profile from GSK

2.1.2 EMOD DTK Summary of methods and resulting vaccine properties

EMOD DTK identified the best-fitting properties for the 3-dose and 4-dose vaccine administrations using a three-step procedure as outlined below. Where mentioned clinical incidence or clinical counts refers to the three-month aggregated intention-to-treat dataset.

Step 1: Establishing effective EIRs for each of the RTS,S trial sites by fitting the clinical incidence in the control arm. Approximate seasonality profiles, or rather normalized seasonality “shapes”, for each site were generated using trial data of clinical incidence as a function of month of the year. We accounted for the distribution of months of the year in which vaccines were administered in each site, also based on trial records, to weight the time-shifted seasonality profiles into an aggregated normalized seasonality curve. We then fit a multiplier, an effective annual EIR, to the clinical incidence data by minimizing the difference between case counts in the trial and in simulations; here, ‘effective’ refers to the fact that we have not explicitly modeled malaria interventions, such as bed nets or indoor residual spraying, due to the paucity of information on coverage by intervention type. (It is also assumed that there are no changes to the implementation of such interventions during the course of the trial.) It is assumed that access to treatment is high in the trial sites, that 90% of clinical cases receive medical care within three days of falling ill. This assumption is supported by the very low rates of severe malaria and malaria-attributed deaths, even in the control arm of the trial. Treatment rates are taken to be the same for both severe and uncomplicated malaria.

Step 2: Fitting the properties of the initial three vaccine doses by comparing the primary clinical incidence in R3 and control arms of the trial. For each individual trial site, using the effective EIR determined in Step 1, the vaccine effect was simulated assuming a vaccine efficacy against infection described by a single exponential curve, parameterized by an initial (or maximum) efficacy and a half-life of protection. As for the fitting of the effective EIR, calendar dates of vaccine administration and site average age at first vaccination were used to weight simulations for alignment with true seasonal malaria exposure.

Simulations were run across a range of initial efficacies and half-lives and Poisson regression was performed to compute the likelihood for the relationship between simulated clinical case counts and trial data for each efficacy-half-life combination. From these maps, the site-specific vaccine best fit was selected by identifying the vaccine properties that yielded the highest likelihood.

Log likelihood maps for the individual trial sites were added to generate a likelihood map for the fit across all sites. The maximum likelihood according to this all-site likelihood map was selected to define the overall best fit vaccine properties. This procedure was conducted separately for the 6-12 week and 5-17 month trial cohorts.

Step 3: Fitting the properties of the 4th dose by comparing the primary clinical incidence in R3R and R3C arms of the trial. The protection against infection of the 4th dose, as with the initial three vaccine doses, was assumed to follow a single exponential curve, though with an independent initial efficacy and half-life. The combined effect of the first three doses and the 4th dose is then functionally described by the sum of two single exponentials.

For each site, simulations were run assuming the site-specific effective EIR and site-specific best fit vaccine properties for the first three doses, across a range of initial efficacies and half-lives for the 4th dose. Likelihood maps were computed for the individual sites, from which the site-specific best fit 4th dose properties were identified. The logs of the individual maps were added to produce the all-site likelihood map from which the best fit 4th dose properties were selected. As before, the procedure was conducted separately for the 6-12 week and 5-17 month trial cohorts.

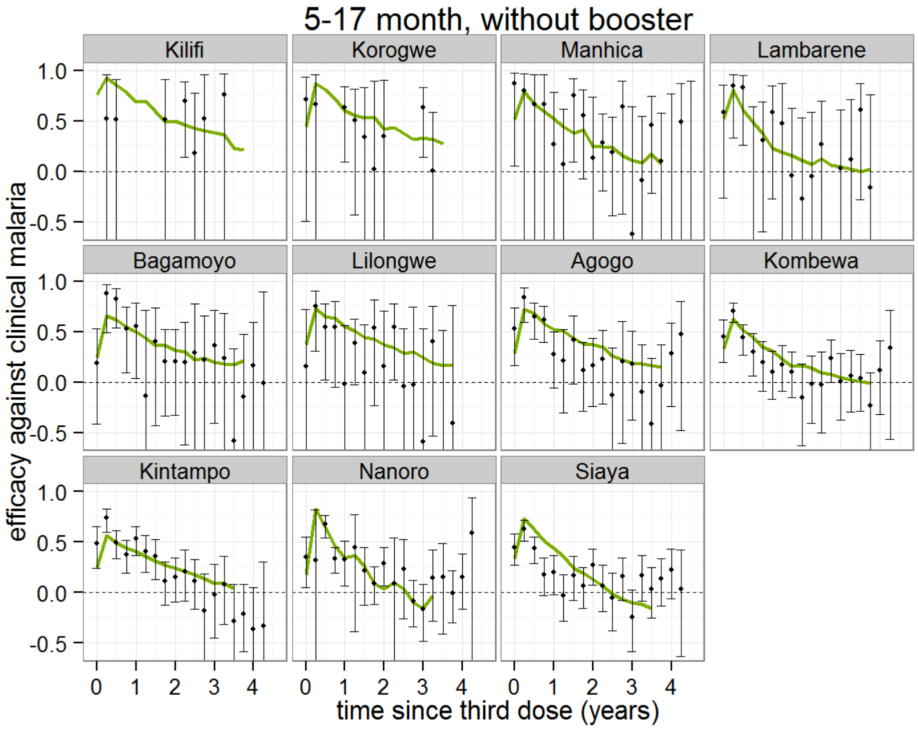

Figure S2.4 Predicted efficacy against clinical disease by trial site compared to Phase III reported for the 5-17 month cohort using best-fitted vaccine profile from EMOD DTK

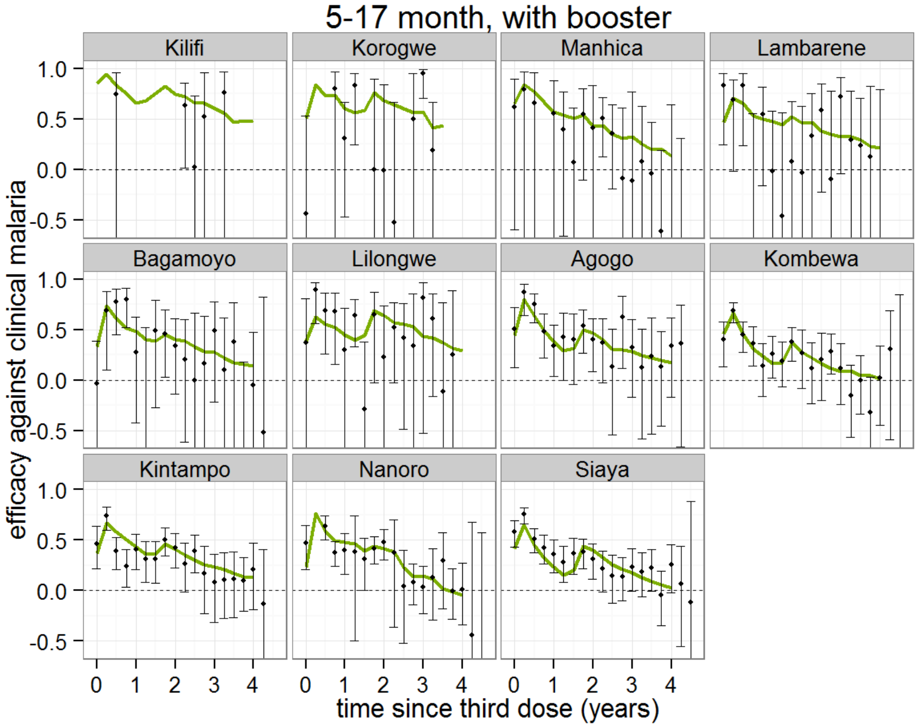

Figure S2.5 Predicted efficacy against clinical disease by trial site compared to Phase III reported for the 5-17 month cohort with boost (4th dose) using best-fitted vaccine profile from EMOD DTK

2.1.3 Imperial Summary of methods and resulting vaccine properties

The vaccine efficacy profile of RTS,S against infection and clinical malaria was estimated in a three step procedure. Firstly, the dynamics of RTS,S induced anti-CSP antibody titres were captured using a model with a bi-phasic pattern of exponential decay. Secondly, the antibody titre at a given time was used to predict efficacy against infection using an estimated dose-response relationship. Thirdly, the vaccine

efficacy against infection was related to efficacy against clinical malaria using a previously validated model for the age and exposure dependent acquisition of clinical immunity. The models were fitted to data from both the 5 to 17 month cohort and the 6 to 12 week cohort. The model was fitted to data from all trial arms (C3C, R3C and R3R) simultaneously using the primary case definition of malaria under according-to-protocol (ATP) conditions[42].Here we focus on the results from the 5 to 17 month cohort.

Following vaccination with RTS,S, anti-CSP antibody titres are assumed to increase to CSP_{peak} and then decay over time *t* according to a bi-phasic exponential model as follows:

$$CSP(t) = CSP_{\text{peak}} \left(\rho_{\text{peak}} e^{-r_s t} + (1-\rho_{\text{peak}}) e^{-r_l t} \right)$$

where r_s and r_l are the decay rates of the short-lived and long-lived components of the antibody response, and ρ_{peak} is the proportion of the antibody response that is short-lived. Following a 4th dose at time $t_{4thDose}$ it is assumed that the rate of decay of the short-lived and long-lived components of the antibody response remain the same, but that the proportion of the response that is short-lived $\rho_{4thDose}$ changes. The antibody dynamics can be described by the following equation:

$$CSP(t) = CSP_{\text{4thDose}} \left(r_{\text{4thDose}} e^{-r_s \left(t - t_{\text{4thDose}}\right)} + (1 - r_{\text{4thDose}}) e^{-r_l \left(t - t_{\text{4thDose}}\right)} \right)$$

The antibody dynamics model was fitted using mixed effects methods to capture the natural variation in antibody dynamics between individuals whilst estimating the average value and variance of the immune parameters across the entire cohort of children. The model was fitted using Bayesian Monte Carlo Markov Chain (MCMC) methods.

The change in vaccine efficacy against infection over time is assumed to be determined by the changing anti-CSP antibody titres. The model-predicted antibody titre at time *t* can be used to predict vaccine efficacy via a dose-response curve defined as follows:

$$V(t) = V_{\max} \left(1 - \frac{1}{1 + \left(\frac{CSP(t)}{\beta} \right)^{\alpha}} \right)$$

where V_{max}, α and β are parameters to be estimated.

For each participant, exposure was determined using a prior EIR for each site combined with a fitted seasonal profile determined from the distribution of cases in the control cohort in each site over calendar time. Heterogeneity in exposure was captured using a Gamma distribution. The probability of clinical disease was then determined from the fitted antibody profile and a model of the relationship between EIR and clinical disease adapted to be consistent with the transmission model described in Griffin et al[12]. This fitting stage was also undertaken in a Bayesian framework using MCMC methods.

Table S2.2: Imperial estimates of parameters describing the dynamics of RTS,S induced anti-CSP antibodies and the dose-response relationship.

Parameter	Description	Prior	Posterior
d_s	half-life of short-lived antibodies	46.0 (44.5, 47.5)	46 (44, 47) days
d_l	half-life of long-lived antibodies	572 (269, 1045)	583 (548, 622) days
ρ_{peak}	proportion of short-lived antibodies following first 3 doses	0.83 (0.63, 0.95)	0.88 (0.87, 0.89)
ρ_{boost}	proportion of short-lived antibodies following 4th dose	0.83 (0.63, 0.95)	0.70 (0.68, 0.72)
σ_s	standard deviation in half-life of short-lived antibodies	U(0, 5000)	16 (12, 25) days
σ_l	standard deviation in half-life of long-lived antibodies	U(0, 5000)	228 (192, 271) days
$\sigma_{\rho,peak}$	standard deviation in proportion short-lived antibodies following first 3 doses	U(0, 5000)	0.10 (0.09, 0.11)
$\sigma_{\rho,4thDose}$	standard deviation in proportion short-lived antibodies following 4th dose	U(0, 5000)	0.19 (0.17, 0.20)
β	dose-response scale parameter	29.1 (6.1, 82.2)	87.3 (63.3, 107.7)
α	dose-response shape parameter	0.92 (0.27, 2.19)	0.77 (0.64, 0.91)
V_{max}	maximum efficacy against infection	0.91 (0.74, 0.99)	0.90 (0.81, 0.97)

Priors and posteriors are presented as median and 95% credible intervals. Prior distributions were informed by results from Phase 2 trials of the RTS,S/AS01 and RTS,S/AS02[25]. U denotes a uniform distribution. Gamma priors were assumed for ds, dl, β and α. Beta priors were assumed for ρ_{peak}, $\rho_{4thDose}$ and V_{max}. Note that the mean and median of a distribution are not necessarily equal.

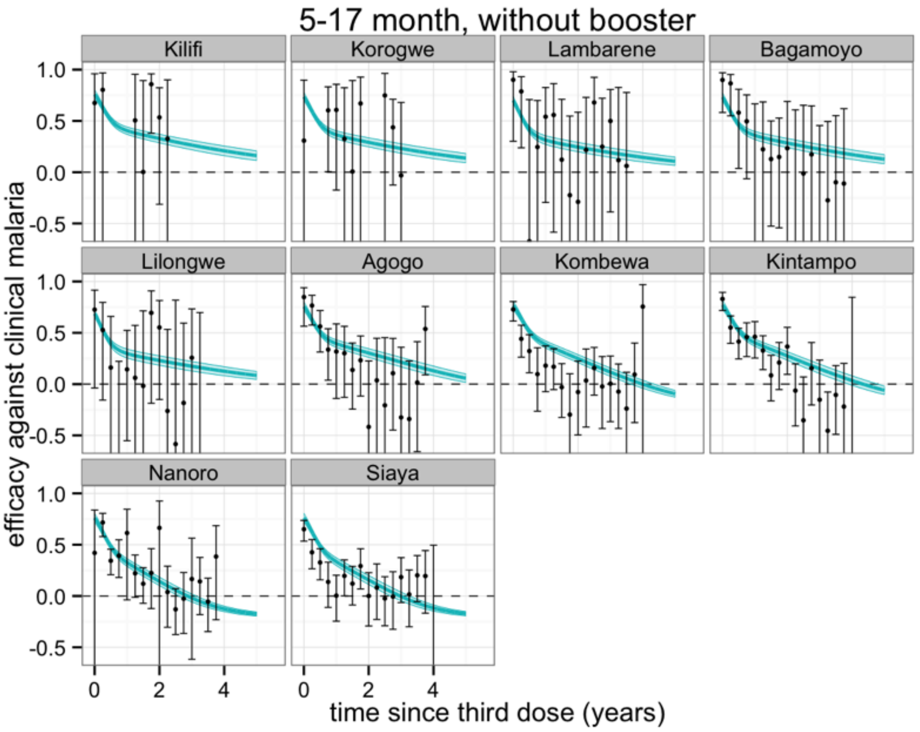

Figure S2.6 Predicted efficacy against clinical disease by trial site compared to Phase III reported for the 5-17 month cohort using best-fitted vaccine profile from Imperial

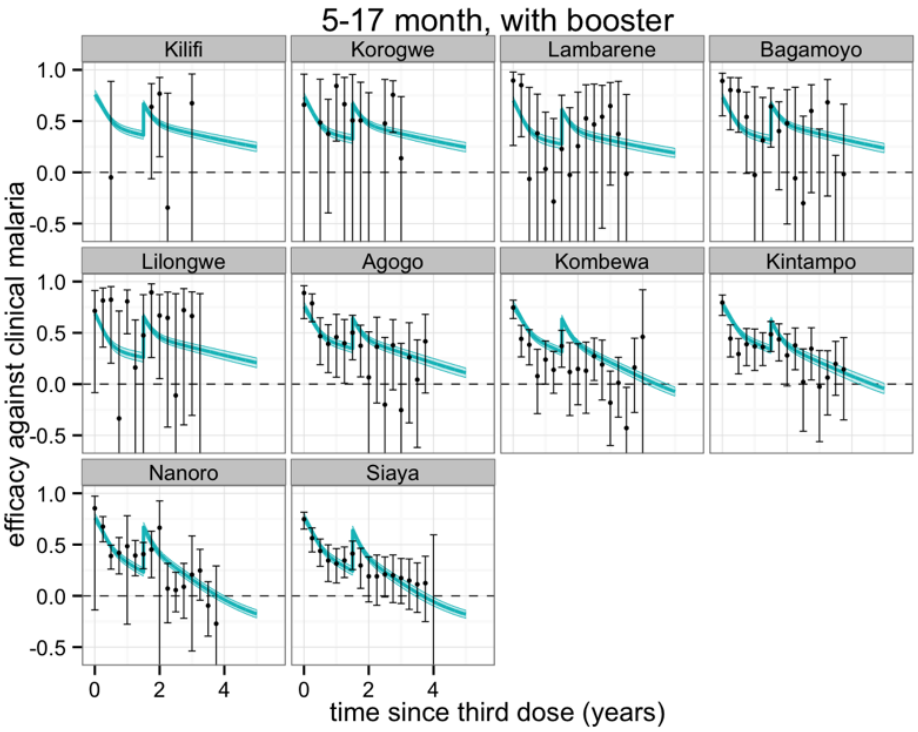

Figure S2.7 Predicted efficacy against clinical disease by trial site compared to Phase III reported for the 5-17 month cohort with boost (4th dose) using best-fitted vaccine profile from Imperial

2.1.4 OpenMalaria Summary of methods and resulting vaccine properties

The vaccine efficacy against infection profile of RTS,S was estimated as previously done for analysis of the 18 month follow-up of the Phase III trial[43]. Each arm of the trial (boost, non-boosting and control for the 5-17 month cohort) was explicitly simulated as an ensemble of 6 models[18]. Using Bayesian MCMC methods, comparing simulated incidence and Phase III trial incidences, the efficacy profile (initial efficacy against infection following the primary schedule of three doses, half-life of decay of efficacy against infection, shape parameters describing the waning profile, and efficacy against infection following a boosting dose, for each cohort was determined. Models were simultaneously fit to the control and vaccinated incidence from each trial site for the primary case definition using aggregated intention-to-treat (ITT).

OpenMalaria allows different rates of decay[44] in underlying efficacy against infection. For this work waning of the efficacy was described by a Weibull decay function curve described by the initial value of the efficacy ε_0, the half-life L, and a shape parameter, k. The Weibull decay function takes the form

$$\varepsilon(t) = \varepsilon_0 exp\left(\frac{-(log2)^{1/k}t}{L^k}\right),$$

where $\varepsilon(t)$ is the efficacy against infection at time t. When $k = 1$, an exponential decay of efficacy against infection is obtained. If k is less than 1, the initial decay is faster than exponential and then slower than exponential after the time equivalent to half-life is reached, this is similar to a bi-phasic like decay, with a sharp decline (quick decay) in efficacy followed by longer decay. For k greater than 1 we observe slow decay of efficacy against infection until the time equivalent to half-life L, and then a much faster decay.

The fitting was implemented as follows:

Step 1: Exposure as a distribution of Entomological Inoculation Rates (EIR) in each trial site was determined using the corresponding control incidence at 3 monthly time points, prevalence in 2-10 year olds recorded at baseline and distribution of prevalences in 2-10 years olds obtained from the MAP 2010.

This distribution of EIR is the EIR profiles that results in predicted prevalences that match the MAP 2010 prevalences with access to treatment in that geographic area imputed from DHS). For fitting, prevalences were scaled to match those observed in the trial sites, namely those correspond to observed high levels of treatment, high LLIN usage, and low severe case incidence. Site distributions of EIR assume current intervention coverage is static and at the level observed in the trial site.

Step 2: A database of simulations from Open Malaria was created, that made both baseline and vaccine impact predictions over a range of EIR, levels of effective case management, and for many hypothetical vaccine efficacy profiles. One database was constructed for each of 6-12 weeks cohorts without and with the 4th dose, 5-17 month cohorts without and with the 4th dose, and the no-vaccine cohort.

Step 3: Using Bayesian MCMC the vaccine efficacy profiles were determined by simultaneously fitting to each trial site observed vaccine incidence and control incidence for that cohort. Case management was allowed to vary to reflect high levels of treatment. Resulting posterior distributions of initial efficacy, half-life and Weibull decay function shape parameter were obtained.

Step 4: The 4th dose efficacy was obtained assuming the same waning profile as the primary course. It was estimated by simultaneously fitting to each trial site observed vaccine incidence in the R3R and C3C cohorts via Bayesian MCMC.

Key results are presented in Table S2.1. For the 5-17 month cohort the fitted vaccine profile was one of high initial efficacy against infection (91%), with a biphasic like decay (shape parameter k =0.69), with half-life approximately 7.3 months and when a 4th dose included the initial efficacy was approximately 50%.

Predicted clinical efficacy (which was not the data used for fitting) by trial site are shown in Figures S2.8 and S2.9.

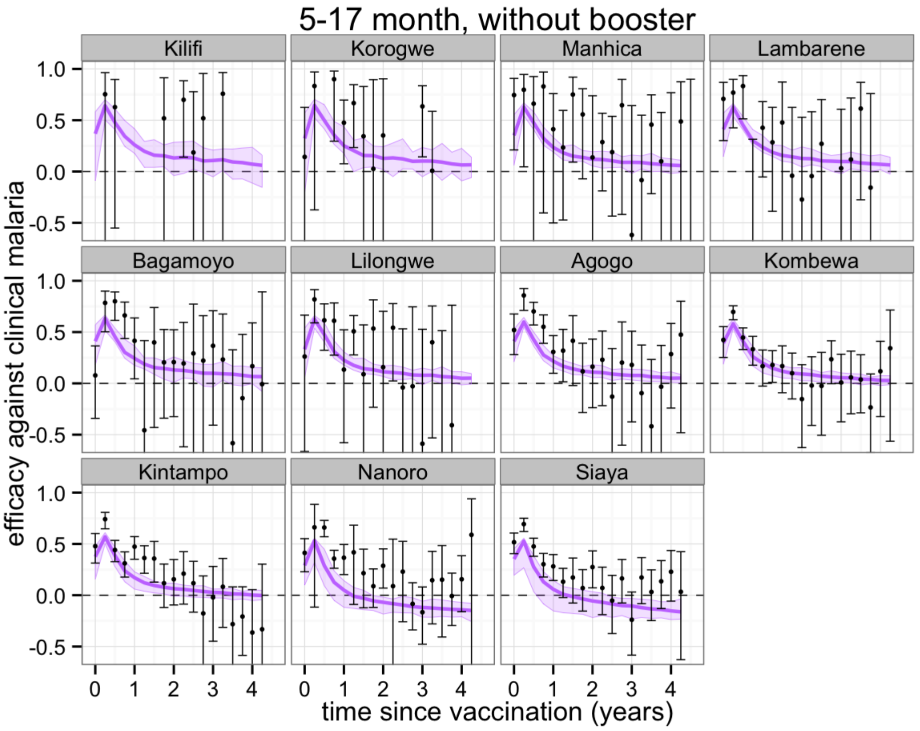

Figure S2.8 Predicted efficacy against clinical disease by trial site compared to Phase III reported for the 5-17 month cohort using best-fitted vaccine profile from OpenMalaria

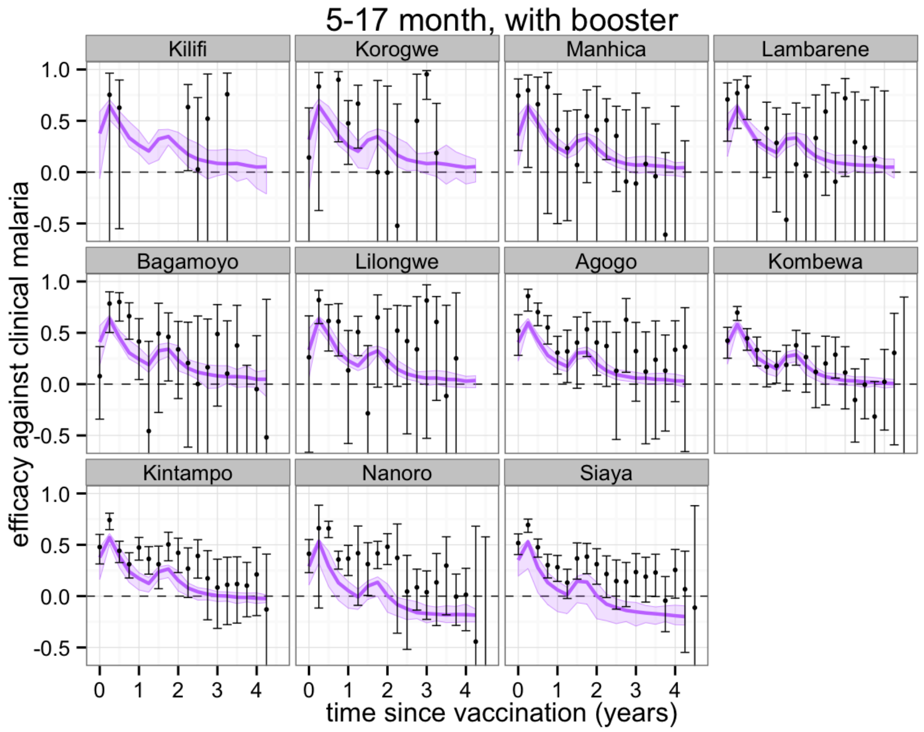

Figure S2.9 Predicted efficacy against clinical disease by trial site compared to Phase III reported for the 5-17 month cohort with boost (4th dose) using best-fitted vaccine profile from OpenMalaria.

3 Cost estimates for economic analysis of RTS,S

3.1 Program costs

Costs of vaccine introduction are calculated from the provider perspective and are limited to cost of consumables including vaccines, injection and reconstitution syringes, safety box; where appropriate, prices are scaled up to account for freight and wastage.

We opt for the limited scope of costing for the transmission scenario analysis to avoid misinterpretation of estimates (heterogeneity in cost of service delivery across countries and transmission intensities); the assumption is justified by previous analyses that showed program costs to be a minor driver of cost-effectiveness estimates for a malaria vaccine deployed routinely[45,46]. The same unit cost is used across transmission profiles and deployment modalities. Costs are reported in 2013 USD. See Table S3.1 for unit prices, quantities, and data sources. Cost per dose is shown under alternate vaccine price assumptions in Table S3.2. As such there is no difference between the cost per visit for a routine schedule (e.g. 6 month and 9 months) and for those outside a routine schedule.

We assumed no drop-off between doses at 90% coverage in the simulated cohort of infants. Thus the number of vaccines is estimated by multiplying the immunized cohort times 3. We assumed a 20 percentage point drop-off for the 4th dose; for this modality number of vaccines is calculated as above for the first 3 doses plus 80% of the immunized cohort for 4th dose.

Total program costs are calculated by multiplying the number of vaccines times the unit cost.

Table S3.1: Prices, quantities, and unit costs for calculating cost of vaccine introduction

Input	Price	Quantity	Unit cost	Source	Notes
**Vaccine**	$5	1	$5	Assumption	$2, $10 per dose used in sensitivity analysis
**Wastage**	10%		$0.56	[45]	
**Freight and insurance**	15%		$0.83	[45]	Applied to vaccine price including wastage
**Injection syringe (0.05ml)**	$0.05	1	$0.05	[46]	-Median supplier price;<br>-Freight included in cost of goods cited by the UNICEF PSQ
**Reconstitution syringe (2ml)**	$0.03	2	$0.06	[46]	-Median supplier price;<br>-Freight included in cost of goods cited by the UNICEF SPQ
**Wastage**	10%		$0.01	[45]	
**Safety box**	$0.60	0.01	$0.01	[46]	-Median supplier price;<br>-100 syringes capacity;<br>-Freight included in cost of goods cited by the UNICEF SPQ
**Wastage**	50%		$0.01	[45]	
**Total per dose**			$6.52		

Table S3.2: Cost per dose administered by vaccine price. Costs capture vaccine and related commodities.

Vaccine price per dose	Unit cost
$5	$6.52
$2	$2.69
$10	$12.91

3.2 Treatment health savings

Costs of malaria case management are calculated from the provider perspective and are limited to cost of diagnostics, antimalarial drugs, and related consumables including syringes, etc.; where appropriate prices are scaled up to account for freight and wastage. Costs of drugs are estimated based on the recommended age dosage and severity of illness. Full compliance with the recommended treatment of malaria, adherence with the drug regimens, and optimal cure rates are assumed for all treatments. Costs are reported in 2013 USD. See Tables S3.3 and S3.4 for unit prices, quantities, and data sources for uncomplicated and severe episodes respectively. Cost of malaria case management by outcome are summarized in Table S3.5. Treatment health savings are estimated by multiplying cost per case averted within each age group times the respective cost per dose.

3.3 Incremental cost-effectiveness ratios

For each deployment modality the incremental cost-effectiveness ratios (ICER) are calculated relative to the baseline case management and for 4th dose implementation additionally relative to the 3-dose schedule. The ratio relates cumulative discounted program costs(3% discounting) net of any health savings realized by the vaccine introduction to the change in health; the latter is expressed in terms of a range of outcomes including DALYs, deaths, episodes, etc. ICERs are reported with and without discounting of benefits at 3%.

Table S3.3: Prices, quantities, and unit costs for calculating cost of uncomplicated malaria episodes

Input	Price	Quantity	Unit cost	Source	Notes
**RDT**	$0.60	1	$0.60	[46]	
**Wastage**	10%		$0.06	Assumption	
**ALU (20mg+120mg, tablet)**	$0.06	12	$0.72	[46]	-Dosage based on weight/age, see Appendix2;<br>- Quantity shown for 5 year old, full course; adjust by age group
**Wastage**	10%		$0.08	Assumption	
**Total per case**			$1.47		

Table S3.4: Prices, quantities, and unit costs for calculating cost of severe malaria episodes

Input	Price	Quantity	Unit cost	Source	Notes
**RDT**	$0.60	1	$0.60	[46]	-Pre-referral testing
**IV Artesunate (60mg, vial)**	$1.83	3	$5.49	[46]	-3 days for recovery, 2 days for death, 7 days for neurological sequilae<br>-Dosage age/ weight specific (see Appendix2)<br>-Quantity shown for 5 year old; adjust by age group
**Injection syringe (10 ml)**	$0.06	3	$0.18	[46]	
**Reconstitution syringe (10 ml)**	$0.06	6	$0.36	[46]	-1 for reconstitution, 1 for dilution
**Dextrose (5%) and Isotonic Saline (0.9%) (1000ml, bottle)**	$1.88	2	$3.76	[46]	-Assuming fluids are needed for the first 2 days<br>-Dosage age/ weight specific (see Appendix2)<br>- Quantity shown for 5 year old; adjust by age group
**Cannula**	$0.15	1	$0.15	[46]	1 per hospitalization
**IV set**	$1.73	2	$3.46	[46]	-1 per hospitalization<br>-Assuming fluids are needed for the first 2 days
**Transfusion set**	$0.33	0.1	$0.03	[46]	-1 per hospitalization -Assigned proportionally with prevalence of severe anaemia; assume 10% of severe cases
**ALU (20mg+120mg, tablet)**	$0.06	12	$0.72	[46]	-Dosage age/ weight specific (see Appendix2)<br>-Quantity shown for 5 year old, full course; adjust by age group
**Paracetamol (100mg, tablet)**	$.01	10	$0.10	[46]	-Dosage age/ weight specific (see Appendix2)<br>- Quantity shown for 5 year old; adjust by age group
**Diazepam (5mg)**	$0.01	0.3	$0.00	[46]	-Dosage age/ weight specific (see Appendix2)<br>-Age/weight dose scaled by probability of convulsions (30%)<br>- Quantity shown for 5 year old, full course; adjust by age group
**Amoxicillin (250mg, tablet)**	$0.02	21	$0.42	[46]	-Dosage age/ weight specific (see Appendix2)<br>- Quantity shown for 5 year old, full course; adjust by age group
**Wastage**	10%		$1.63	Assumption	
**Microscopy**	$1.5	4	$6.0	Review	Daily
**Safety box**	$0.60	0.09	$0.05	[46]	
**Wastage**	50%		$0.05	[45]	
**Cost per severe case**			$22.41		

Table S3.5: Cost of malaria case management by age group and severity of illness (USD, 2013). Malaria case management costs cover antimalarials and related medication and supplies, including freight and wastage.

		**Cost per severe case by outcome**			
**Age Group**	**Cost per uncomplicated case**	**Recovery**	**Neurological Sequilae**	**Death**	**Any outcome**
0-1	$1.07	$22.13	$31.20	$16.46	$21.78
1-2	$1.07	$22.28	$31.36	$16.61	$21.93
2-3	$1.07	$23.71	$32.79	$17.82	$23.35
3-4	$1.47	$24.19	$33.26	$17.90	$23.79
4-5	$1.47	$24.41	$33.49	$18.12	$24.02
5-10	$1.47	$28.82	$37.90	$22.53	$28.43
10-12	$1.87	$35.93	$53.14	$27.09	$35.43
12-16	$2.27	$43.80	$61.01	$34.45	$43.26
16-20	$2.27	$50.43	$75.77	$39.05	$49.82
20-100	$2.27	$56.53	$90.00	$43.11	$55.85

4 Additional vaccine public health impact and cost-effectiveness results

Here we include additional outputs to support predictions described in the main manuscript.

4.1 DALY calculations

Disability adjusted life years (DALYs) were calculated based on the duration of disability and respective disability weights. Weights by disease outcome and treatment have been obtained from the Global Burden of Disease study[47]. Life-time disability is assumed for severe episodes that result in neurological sequelae. Years of life lost (YLLs) and DALYs were calculated assuming age-specific life expectancies, based on the life-table from Butajira, Ethiopia, with an average life expectancy at birth of 46.6 years[48]. YLLs and DALYs were estimated based on a comprehensive measure of deaths that includes both direct malaria deaths and deaths due to malaria co-morbidities. For comparison, estimates based on direct malaria deaths only are also reported. In the light of the recent revised recommendations[49] DALYs are presented without age-weighting and discounting. For comparison with previous estimates and given lack of a general consensus in the literature we also report DALYs based on discounted health benefits at 3% in the Appendix tables.

4.2 Additional Results

4.2.1 Vaccine public health impact

Cumulative impact of RTS,S is reported at year 15 after the vaccine introduction and is summarized in terms of clinical malaria cases, severe cases, hospitalised cases, deaths, and DALYs. Age specific estimates are provided in Figures S4.1 to S4.4 for both 3 and 4-dose schedules. Furthermore, age aggregated illustrations of the predicted number of events averted by the RTS,S per 100,000 fully vaccinated children are shown in Figure S4.5 for the schedule with a 4th dose (without 4th dose not shown).

For *Pf*PR$_{2\text{-}10}$ below 10%, a positive impact was predicted by all models, however, at 3% *Pf*PR$_{2\text{-}10}$ the uncertainty intervals include zero (Tables S4.1- S4.2). There is more divergence in median predictions between the models in low (*Pf*PR$_{2\text{-}10}$ < 10%) compared to higher transmission settings (*Pf*PR$_{2\text{-}10}$ of 10% to 65%), however, confidence intervals overlap. For *Pf*PR$_{2\text{-}10}$ above 3% up to 10% the impact is positive. At *Pf*PR$_{2\text{-}10}$ of 5%, the models estimate 129 (74-178) deaths are averted for every 100,000 fully vaccinated via the 6-9 month schedule and 144 (102-249) deaths are averted for every 100,000 fully vaccinated with the 6-9 month schedule with a 4th dose. This averts 21% (8-25%) of malaria deaths in children under five via the 6-9 month schedule and 28% (9-31%) of malaria deaths in children under five via a 4-dose schedule. The same numbers for *Pf*PR$_{2\text{-}10}$ of 7.5%, are 187.5 (106-251) deaths are averted for every 100,000 fully vaccinated via the 6-9 month schedule and 224 (147-305) deaths are averted for every 100,000 fully vaccinated with the 6-9 month schedule with a 4th dose. This averts 19.9% (8.6-21.9) of malaria deaths in children under five via the 6-9 month schedule and 26.15% (9.1-27.8%) of malaria deaths in children under five via a 4-dose schedule.

Partially protective malaria interventions reduce an individual's exposure to malaria infection and the subsequent effect is to delay acquisition of natural immunity in those individuals compared to non-intervened[23,50]. A delay of blood-stage immunity acquisition is predicted and observed to result in a shift of clinical and severe disease to older ages[23,50-53]. Exposure to malaria infection at older ages leads to higher rates of disease in all models in individuals newly exposed compared to those individuals experiencing the same force of infection earlier in life. This has been observed in numerous trials and investigated in settings with patterns of decreasing transmission during interventions; with the age-shift either reduced or prevented[23,50,53]. This age-shift is more dramatic in higher prevalence settings where natural immunity is acquired more rapidly. Combining this effect with the estimated biological waning of the vaccine, and

assuming force of infection within the population remains at the same levels over time, means that some of the initial impact of the vaccine on the cases averted in very young children is predicted to be offset by higher relative incidence at older ages (Figure S1.1 and Figures S4.1-S4.4), with this effect predicted to be delayed by a schedule which includes a 4th dose. Similar effects are also predicted for severe disease, with the age-shift occurring earlier than for clinical cases (Figures S4.1-S4.4), and predicted in the Phase III trial for the 3-dose schedule[54].

The absolute burden of the disease prevented through routine use of RTS,S is predicted to increase up to *Pf*PR$_{2\text{-}10}$ of 50-65%. At higher prevalence, while the predictions of the models diverge, the absolute impact either reaches a plateau or decreases (Figure S4.5); the greatest decrease in impact is predicted by OpenMalaria.

Variation in predictions should be interpreted in the context of differences between the models. In particular, GSK and EMOD DTK differ from the OpenMalaria and Imperial in not including of the potential indirect (herd) effects of vaccination. Furthermore, the different mechanisms that the models use to translate efficacy against infection into efficacy against clinical disease contribute to variation in predictions.

4.2.2 Cost effectiveness

Incremental cost-effectiveness ratios (ICERs) estimated using cumulative outcomes at year 15 of the program for 3- and 4-dose deployments are shown in Figure 4 in the main text. These data are summarized in tabular form in Table S4.3 and Table S4.5. The estimates are also represented as slopes on incremental cost-effectiveness planes for the 5 transmission intensity settings assuming vaccine cost per dose of either USD$2, 5 or 10 in Figure S4.6.

The ICERs for the simulated vaccination programs are generally in good agreement between the four models; lowest cost-effectiveness ratios are predicted by GSK and highest by EMOD DTK . For all models the lowest ICER is predicted at intermediate levels of transmission (*Pf*PR$_{2\text{-}10}$ of 30-50%) predicted $43-100 per DALY averted assuming a price of $5 per dose (or US$18-45 per DALY averted assuming $2 per dose). The predicted incremental benefit of adding a 4th dose to the primary schedule differs between the models, with OpenMalaria predicting a minimal additional benefit for the 4th dose (related to boosting efficacy against infection profiles). Consequently, while GSK, Imperial, and EMOD DTK all predict somewhat more favourable cost-effective ratios for a 4th dose, predictions by OpenMalaria suggest that the added benefit is offset by the higher cost of the program implementation.

For *Pf*PR$_{2\text{-}10}$ below 10% there is less agreement between the models, with at one extreme Imperial predicting small increases to the ICERs, compared to predicted large increase in ICERs by EMOD DTK.

Table S4.1: Deaths averted per 100,000 fully vaccinated and percentage of under 5 deaths averted by RTS,S for 6-9 month immunization schedule with or without 4th dose at 15 years follow-up for representative parasite prevalence settings (*Pf*PR_{2-10}). Estimates are medians and 95% prediction interval for each model.

	PfPR_{2-10} = 3%	*Pf*PR_{2-10} = 10%	*Pf*PR_{2-10} = 30%	*Pf*PR_{2-10} = 50%	*Pf*PR_{2-10} = 65%
**Deaths averted per 100,000 fully vaccinated by 6-9 month immunization schedule**					
EMOD DTK	43 (26 to 71)	127 (79 to 194)	287 (111 to 561)	473 (187 to 708)	685 (357 to 1052)
GSK	63 (-5 to 134)	210 (77 to 339)	570 (347 to 802)	708 (338 to 1055)	-
Imperial	136 (9 to 267)	251 (132 to 394)	391 (222 to 594)	429 (226 to 748)	397 (105 to 862)
OpenMalaria	66 (17 to 150)	200 (97 to 300)	394 (258 to 580)	496 (328 to 682)	350 (207 to 550)
**Percentage of malaria deaths averted in children younger than 5 years by 6-9 month immunization schedule**					
EMOD DTK	23.8 (13.5 to 30.5)	20.9 (16.4 to 25)	17.7 (10.3 to 27.2)	16.6 (10.4 to 21.6)	16 (9.8 to 19.8)
GSK	24.3 (-5.3 to 45.3)	21.4 (11.8 to 32.3)	15.5 (10.6 to 20.2)	13.8 (8.9 to 18.7)	-
Imperial	22.3 (8.7 to 26.4)	18.1 (14.9 to 22.2)	13.7 (10.9 to 17.8)	11.1 (7.7 to 15.7)	9.9 (4.4 to 15.9)
OpenMalaria	7.8 (4.1 to 14.4)	9.8 (6.8 to 12.9)	10.8 (8.6 to 13)	9 (7.3 to 10.6)	5.3 (4.3 to 7.6)
**Deaths averted per 100,000 fully vaccinated by 6-9 month immunization schedule with 4th dose**					
EMOD DTK	61 (39 to 83)	189 (142 to 284)	406 (205 to 643)	554 (333 to 828)	838 (454 to 1180)
GSK	81 (28 to 150)	254 (139 to 383)	715 (503 to 953)	859 (571 to 1197)	-
Imperial	184 (46 to 368)	344 (182 to 539)	501 (294 to 749)	528 (293 to 922)	484 (144 to 991)
OpenMalaria	61 (25 to 144)	205 (118 to 316)	417 (308 to 562)	540 (326 to 663)	376 (223 to 553)
**Percentage of malaria deaths averted in children younger than 5 years by 6-9 month immunization schedule with 4th dose**					
EMOD DTK	29.7 (23.2 to 35.9)	29.1 (25.1 to 32.8)	26.3 (19.6 to 33)	20.2 (16.1 to 27.3)	19.5 (12.4 to 24)
GSK	33.3 (11.5 to 47)	26.7 (16.4 to 34.7)	20.7 (16.1 to 24.7)	18 (12.7 to 22.4)	-
Imperial	28.7 (17.4 to 33.7)	23.5 (20.1 to 27.4)	17.5 (14 to 22)	14 (10.4 to 18.9)	12.2 (6.4 to 18.1)
OpenMalaria	8.9 (5.3 to 14.3)	10.3 (7.5 to 13.3)	11.4 (9.5 to 13.3)	9.7 (7.4 to 10.6)	6 (4.6 to 7.6)

Table S4.2: Clinical cases averted per 100,000 fully vaccinated and proportion of clinical cases averted by RTS,S for 6-9 month immunization schedule with or without 4th dose at 15 years follow-up for representative transmission settings (*Pf*PR$_{2-10}$). Estimates are medians and 95% prediction interval for each model.

	***Pf*PR$_{2-10}$ = 3%**	***Pf*PR$_{2-10}$ = 10%**	***Pf*PR$_{2-10}$ = 30%**	***Pf*PR$_{2-10}$ = 50%**	***Pf*PR$_{2-10}$ = 65%**
**Clinical cases averted per 100,000 fully vaccinated by 6-9 month immunization schedule**					
EMOD DTK	5771 (5180 to 6096)	20491 (19936 to 21428)	61553 (56026 to 66316)	93938 (86001 to 102252)	98877 (89804 to 114327)
GSK	6744 (5570 to 7989)	27877 (25618 to 30284)	106031 (95597 to 115489)	126545 (105380 to 144657)	-
Imperial*	14944 (-16912 to 31958)	39446 (23064 to 77032)	84590 (51532 to 126304)	114655 (71191 to 164880)	120178 (74129 to 181458)
OpenMalaria	14889 (13860 to 15791)	47542 (43628 to 49806)	107702 (96618 to 112579)	116689 (101692 to 130131)	50705 (13036 to 72007)
**Proportion of clinical cases averted in under five year olds by 6-9 month immunization schedule**					
EMOD DTK	20.4% (19.5 to 21.3)	19.7% (19.2 to 20.3)	18.6% (17.6 to 19.5)	16.4% (15.7 to 16.8)	13.4% (12.7 to 13.9)
GSK	26.6% (23.2 to 30)	24.1% (22.4 to 25.7)	20% (18.9 to 20.8)	16.2% (15 to 17.4)	-
Imperial	23.8% (9 to 28.3)	21.7% (18.5 to 25.9)	18.7% (16 to 23.2)	15.8% (13.3 to 19.8)	13.5% (11 to 18.1)
OpenMalaria	15.9% (15.5 to 16.2)	15.6% (15.1 to 15.8)	14.1% (13.6 to 14.2)	11.9% (11.1 to 12.1)	7.3% (4.8 to 8.3)
**Clinical cases averted per 100,000 fully vaccinated by 6-9 month immunization schedule with 4th dose**					
EMOD DTK	8769 (8227 to 9228)	31448 (30478 to 32257)	93609 (88791 to 97926)	134415 (127719 to 151866)	139374 (122550 to 159521)
GSK	8579 (7540 to 9446)	35143 (32621 to 37762)	134974 (124847 to 142563)	160411 (141739 to 179734)	-
Imperial*	21455 (-8358 to 46127)	55760 (32647 to 98616)	116482 (73731 to 166219)	154606 (96978 to 210814)	160236 (102561 to 231577)
OpenMalaria	14659 (13664 to 15418)	46978 (43285 to 48971)	108824 (96821 to 113330)	121182 (104238 to 133815)	55849 (15613 to 77045)
**Proportion of clinical cases averted in under five year olds by 6-9 month immunization schedule with 4th dose**					
EMOD DTK	29.2% (28.5 to 30.3)	28.4% (28 to 28.9)	27% (26 to 28)	23.3% (22.8 to 23.9)	18.5% (18 to 19.2)
GSK	33.5% (30.5 to 35.9)	30.6% (29.2 to 31.9)	25.7% (24.9 to 26.7)	21.1% (20.1 to 22.5)	-
Imperial	30.7% (18.1 to 35.5)	28.1% (24.6 to 32)	24.4% (21.5 to 29.2)	20.6% (18 to 24.2)	17.8% (15.3 to 22.3)
OpenMalaria	16.7% (16.2 to 16.9)	16.4% (15.9 to 16.6)	14.9% (14.3 to 15)	12.6% (11.8 to 12.9)	7.9% (5.3 to 8.9)

*****Negative cases averted at low transmission are due to stochastic variation between model runs at low prevalence rather than due to any modelled biological mechanism.

Table S4.3: Cost per DALY averted by RTS,S for 6-9 month immunization schedule with or without 4th dose at 15 years follow-up for representative parasite prevalence settings (*Pf*PR_{2-10}). Estimates are medians and 95% prediction interval for each model.

	PfPR_{2-10} = 3%	*Pf*PR_{2-10} = 10%	*Pf*PR_{2-10} = 30%	*Pf*PR_{2-10} = 50%	*Pf*PR_{2-10} = 65%
**Cost per DALY averted (USD$) by 6-9 month immunization schedule ($2 a dose)**					
EMOD DTK	$312 ($193 to 475)	$112 ($73 to 161)	$29 ($16 to 57)	$21 ($13 to 32)	$16 ($9 to 25)
GSK	$157 ($-2067 to 1199)	$54 ($33 to 371)	$19 ($13 to 33)	$18 ($11 to 62)	-
Imperial	$87 ($-48 to 211)	$55 ($34 to 102)	$43 ($27 to 78)	$45 ($24 to 89)	$50 ($21 to 185)
OpenMalaria	$181 ($76 to 31347)	$58 ($33 to 177)	$28 ($19 to 46)	$24 ($16 to 42)	$35 ($22 to 80)
**Cost per DALY averted (USD$) by 6-9 month immunization schedule ($5 a dose)**					
EMOD DTK	$763 ($471 to 1159)	$279 ($183 to 399)	$77 ($42 to 148)	$56 ($37 to 86)	$44 ($26 to 67)
GSK	$383 ($-5038 to 2925)	$134 ($82 to 919)	$51 ($34 to 88)	$49 ($30 to 161)	-
Imperial	$205 ($-107 to 480)	$117 ($74 to 222)	$82 ($54 to 147)	$80 ($45 to 155)	$89 ($40 to 336)
OpenMalaria	$439 ($184 to 75820)	$144 ($80 to 433)	$71 ($49 to 114)	$59 ($39 to 103)	$84 ($53 to 190)
**Cost per DALY averted (USD$) by 6-9 month immunization schedule ($10 a dose)**					
EMOD DTK	$1514 ($936 to 2300)	$556 ($366 to 795)	$155 ($86 to 302)	$114 ($77 to 176)	$90 ($53 to 136)
GSK	$761 ($-9989 to 5801)	$269 ($165 to 1833)	$105 ($70 to 178)	$99 ($62 to 324)	-
Imperial	$402 ($-205 to 932)	$219 ($139 to 419)	$147 ($97 to 261)	$139 ($79 to 265)	$154 ($70 to 588)
OpenMalaria	$867 ($364 to 149941)	$286 ($160 to 860)	$142 ($97 to 228)	$118 ($77 to 206)	$166 ($105 to 372)
**Cost per DALY averted (USD$) by 6-9 month immunization schedule with 4th dose ($2 a dose)**					
EMOD DTK	$298 ($213 to 463)	$97 ($66 to 132)	$36 ($21 to 57)	$26 ($17 to 41)	$18 ($12 to 32)
GSK	$148 ($77 to 591)	$53 ($34 to 110)	$18 ($13 to 27)	$18 ($12 to 32)	-
Imperial	$83 ($34 to 187)	$48 ($31 to 88)	$38 ($25 to 69)	$40 ($22 to 73)	$47 ($21 to 146)
OpenMalaria	$250 ($94 to 1491)	$75 ($45 to 132)	$35 ($23 to 53)	$28 ($22 to 51)	$42 ($27 to 82)
**Cost per DALY averted (USD$) by 6-9 month immunization schedule with 4th dose ($5 a dose)**					
EMOD DTK	$728 ($522 to 1130)	$244 ($166 to 328)	$96 ($56 to 149)	$71 ($46 to 110)	$48 ($35 to 85)
GSK	$362 ($190 to 1443)	$133 ($86 to 273)	$49 ($34 to 72)	$49 ($33 to 83)	-
Imperial	$195 ($80 to 440)	$105 ($68 to 195)	$77 ($50 to 133)	$76 ($43 to 136)	$87 ($41 to 278)
OpenMalaria	$602 ($227 to 3596)	$183 ($111 to 323)	$87 ($57 to 131)	$69 ($54 to 126)	$101 ($66 to 195)

Cost per DALY averted (USD$) by 6-9 month immunization schedule with 4th dose ($10 a dose)					
EMOD DTK	$1447 ($1037 to 2243)	$487 ($332 to 657)	$197 ($116 to 303)	$146 ($96 to 224)	$99 ($73 to 173)
GSK	$720 ($377 to 2862)	$267 ($172 to 546)	$100 ($70 to 147)	$100 ($66 to 168)	-
Imperial	$382 ($158 to 861)	$200 ($128 to 374)	$141 ($93 to 241)	$137 ($78 to 245)	$154 ($74 to 500)
OpenMalaria	$1189 ($448 to 7105)	$363 ($221 to 640)	$174 ($114 to 261)	$138 ($107 to 251)	$199 ($131 to 383)

Table S4.4: Deaths averted per 100,000 fully vaccinated and percentage of under 5 deaths averted by RTS,S for 6-9 month immunization schedule with or without 4th dose at 15 years follow-up for low prevalence representative transmission settings (*Pf*PR_{2-10}). Estimates are medians and 95% prediction interval for each model.

	PfPR_{2-10} = 3%	*Pf*PR_{2-10} = 5%	*Pf*PR_{2-10} = 7.5%	*Pf*PR_{2-10} = 10%	*Pf*PR_{2-10} = 15%
**Deaths averted per 100,000 fully vaccinated by 6-9 month immunization schedule**					
EMOD DTK	43 (26 to 71)	74 (46 to 103)	106 (68 to 153)	127 (79 to 194)	160 (99 to 247)
GSK	63 (-5 to 134)	85 (7 to 160)	141 (47 to 217)	210 (77 to 339)	390 (210 to 577)
Imperial	136 (9 to 267)	178 (78 to 306)	225 (110 to 359)	251 (132 to 394)	306 (169 to 472)
OpenMalaria	66 (17 to 150)	115 (29 to 259)	150 (70 to 287)	200 (97 to 300)	266 (164 to 388)
**Percentage of deaths averted in children younger than 5 years by 6-9 month immunization schedule**					
EMOD DTK	23.8% (13.5 to 30.5)	21.6% (15.1 to 27.6)	20.5% (15.7 to 25.4)	20.9% (16.4 to 25)	19.8% (16.5 to 22.7)
GSK	24.3% (-5.3 to 45.3)	27.4% (7.3 to 43.3)	21.5% (9.9 to 32.9)	21.4% (11.8 to 32.3)	16.1% (10.3 to 24)
Imperial	22.3% (8.7 to 26.4)	20.7% (16.7 to 25.4)	19.3% (15.6 to 23.2)	18.1% (14.9 to 22.2)	16.4% (13.7 to 20.2)
OpenMalaria	7.8% (4.1 to 14.4)	7.8% (4.1 to 14.4)	8.6% (6.8 to 13.3)	9.8% (6.8 to 12.9)	11.1% (8.1 to 13.5)
**Deaths averted per 100,000 fully vaccinated by 6-9 month immunization schedule with 4th dose**					
EMOD DTK	61 (39 to 83)	102 (74 to 145)	151 (113 to 203)	189 (142 to 284)	250 (178 to 334)
GSK	81 (28 to 150)	107 (49 to 172)	174 (75 to 258)	254 (139 to 383)	511 (333 to 684)
Imperial	184 (46 to 368)	249 (114 to 430)	297 (157 to 495)	344 (182 to 539)	414 (218 to 603)
OpenMalaria	61 (25 to 144)	106 (43 to 248)	147 (101 to 287)	205 (118 to 316)	293 (170 to 381)
**Percentage of deaths averted in children younger than 5 years by 6-9 month immunization schedule with 4th dose**					
EMOD DTK	29.7% (23.2 to 35.9)	29.1% (23.1 to 35.5)	27.8% (24.4 to 32.1)	29.1% (25.1 to 32.8)	28.8% (25.6 to 31)
GSK	33.3% (11.5 to 47)	32.5% (20.4 to 48.8)	28.5% (18 to 37.3)	26.7% (16.4 to 34.7)	22.1% (14.2 to 26.9)
Imperial	28.7% (17.4 to 33.7)	27% (22.8 to 32.5)	25% (21.2 to 28.8)	23.5% (20.1 to 27.4)	21.4% (18.4 to 25.2)
OpenMalaria	8.9% (5.3 to 14.3)	8.9% (5.3 to 14.3)	9.1% (7.6 to 13.1)	10.3% (7.5 to 13.3)	11.8% (8.9 to 13.7)

Table S4.5: Cost per DALY averted by RTS,S for 6-9 month immunization schedule with or without 4th dose at 15 years follow-up for low prevalence representative transmission settings (*Pf*PR$_{2-10}$). Estimates are medians and 95% prediction interval for each model.

	*Pf*PR$_{2-10}$ = 3%	*Pf*PR$_{2-10}$ = 5%	*Pf*PR$_{2-10}$ = 7.5%	*Pf*PR$_{2-10}$ = 10%	*Pf*PR$_{2-10}$ = 15%
**Cost per DALY averted (USD$) by 6-9 month immunization schedule ($2 a dose)**					
EMOD DTK	$312 ($193 to 475)	$204 ($142 to 300)	$135 ($96 to 213)	$112 ($73 to 161)	$89 ($58 to 139)
GSK	$157 ($-2067 to 1199)	$128 ($-234 to 3530)	$76 ($50 to 376)	$54 ($33 to 371)	$27 ($18 to 68)
Imperial	$87 ($-48 to 211)	$70 ($39 to 154)	$60 ($36 to 122)	$55 ($34 to 102)	$48 ($31 to 87)
OpenMalaria	$181 ($76 to 31347)	$104 ($44 to 17867)	$77 ($38 to 161)	$58 ($33 to 177)	$42 ($29 to 76)
**Cost per DALY averted (USD$) by 6-9 month immunization schedule ($5 a dose)**					
EMOD DTK	$763 ($471 to 1159)	$500 ($349 to 736)	$334 ($237 to 523)	$279 ($183 to 399)	$223 ($147 to 348)
GSK	$383 ($-5038 to 2925)	$314 ($-569 to 8613)	$188 ($123 to 924)	$134 ($82 to 919)	$70 ($46 to 173)
Imperial	$205 ($-107 to 480)	$159 ($88 to 344)	$130 ($78 to 261)	$117 ($74 to 222)	$98 ($65 to 178)
OpenMalaria	$439 ($184 to 75820)	$252 ($106 to 43586)	$190 ($94 to 394)	$144 ($80 to 433)	$105 ($72 to 189)
**Cost per DALY averted (USD$) by 6-9 month immunization schedule ($10 a dose)**					
EMOD DTK	$1514 ($936 to 2300)	$995 ($693 to 1462)	$665 ($474 to 1041)	$556 ($366 to 795)	$446 ($294 to 697)
GSK	$761 ($-9989 to 5801)	$625 ($-1128 to 17086)	$376 ($246 to 1837)	$269 ($165 to 1833)	$142 ($94 to 349)
Imperial	$402 ($-205 to 932)	$306 ($168 to 662)	$246 ($150 to 493)	$219 ($139 to 419)	$181 ($119 to 329)
OpenMalaria	$867 ($364 to 149941)	$500 ($210 to 86451)	$377 ($188 to 781)	$286 ($160 to 860)	$211 ($144 to 376)
**Cost per DALY averted (USD$) by 6-9 month immunization schedule with 4th dose ($2 a dose)**					
EMOD DTK	$298 ($213 to 463)	$188 ($133 to 262)	$126 ($93 to 165)	$97 ($66 to 132)	$72 ($54 to 101)
GSK	$148 ($77 to 591)	$116 ($75 to 364)	$74 ($48 to 272)	$53 ($34 to 110)	$24 ($17 to 39)
Imperial	$83 ($34 to 187)	$62 ($36 to 132)	$53 ($32 to 105)	$48 ($31 to 88)	$42 ($27 to 76)
OpenMalaria	$250 ($94 to 1491)	$144 ($54 to 853)	$101 ($52 to 157)	$75 ($45 to 132)	$51 ($38 to 91)
**Cost per DALY averted (USD$) by 6-9 month immunization schedule with 4th dose ($5 a dose)**					
EMOD DTK	$728 ($522 to 1130)	$462 ($328 to 643)	$312 ($232 to 409)	$244 ($166 to 328)	$181 ($136 to 256)
GSK	$362 ($190 to 1443)	$285 ($186 to 889)	$183 ($120 to 669)	$133 ($86 to 273)	$63 ($45 to 100)
Imperial	$195 ($80 to 440)	$143 ($84 to 305)	$120 ($72 to 230)	$105 ($68 to 195)	$89 ($59 to 165)
OpenMalaria	$602 ($227 to 3596)	$347 ($131 to 2070)	$245 ($127 to 380)	$183 ($111 to 323)	$126 ($95 to 222)

**Cost per DALY averted (USD$) by 6-9 month immunization schedule with 4th dose ($10 a dose)**					
EMOD DTK	$1447 ($1037 to 2243)	$919 ($654 to 1280)	$622 ($463 to 815)	$487 ($332 to 657)	$363 ($275 to 514)
GSK	$720 ($377 to 2862)	$567 ($369 to 1765)	$366 ($240 to 1330)	$267 ($172 to 546)	$128 ($92 to 203)
Imperial	$382 ($158 to 861)	$278 ($163 to 594)	$232 ($139 to 439)	$200 ($128 to 374)	$168 ($114 to 313)
OpenMalaria	$1189 ($448 to 7105)	$686 ($259 to 4099)	$486 ($252 to 753)	$363 ($221 to 640)	$249 ($188 to 442)

Table S4.6: Summary predictions of public health impact and cost-effectiveness of RTS,S for 6-9 month immunization schedule with or without 4th dose at 15 years follow-up. Estimates are presented as median and ranges across the model's medians

Outcome	Vaccination Schedule	*Pf*PR$_{2-10}$ 3% to 65%	*Pf*PR$_{2-10}$ 10% to 65%	*Pf*PR$_{2-10}$ 10% to 50%	*Pf*PR$_{2-10}$ 30% to 50%	*Pf*PR$_{2-10}$ 10%	*Pf*PR$_{2-10}$ 7·5%	*Pf*PR$_{2-10}$ 5%
**Public Health Impact**								
Proportion of deaths under 5 averted	6-9 months with 4th dose	19.5% (6-33.3)	18% (6-29.1)	19.1% (9.7-29.1)	17.8% (9.7-26.3)	25.1% (10.3-29.1)	26.4% (9.1-28.5)	28.1% (8.9-32.5)
	6-9 months	15.5% (5.3-24.3)	13.8% (5.3-21.4)	14.7% (9-21.4)	13.8% (9-17.7)	19.5% (9.8-21.4)	19.9% (8.6-21.5)	21.2% (7.8-27.4)
Proportion of clinical cases under 5 averted	6-9 months with 4th dose	23.3% (7.9-33.5)	21.1% (7.9-30.6)	23.9% (12.6-30.6)	22.2% (12.6-27)	28.3% (16.4-30.6)	28.6% (16.5-31.6)	29.3% (16.7-33)
	6-9 months	16.4% (7.3-26.6)	16.2% (7.3-24.1)	17.5% (11.9-24.1)	16.3% (11.9-20)	20.7% (15.6-24.1)	21% (15.8-25.3)	21.45% (15.9-26.2)
Deaths averted per 100,000 fully vaccinated	6-9 months 4th dose	406 (61-859)	484 (189-859)	459 (189-859)	534 (406-859)	229.5 (189-344)	162.5 (147-297)	106.5 (102-249)
	6-9 months	350 (43-708)	394 (127-708)	392.5 (127-708)	451 (287-708)	205 (127-251)	145.5 (106-225)	100 (74-178)
Clinical cases averted per 100,000 fully vaccinated	6-9 months 4th dose	93609 (8579-160411)	116482 (31448-160411)	112653 (31448-160411)	127799 (93609-160411)	41060.5 (31448-55760)	29740.5 (21799-46784)	20299.5 (11072-34063)
	6-9 months	61553 (5771-126545)	93938 (20491-126545)	89264 (20491-126545)	106867(61553-126545)	33661.5 (20491-47542)	24807 (14790-37273)	16788 (8854-25745)
**Incremental benefit (% of additional events averted of boosting schedule compared to non-boosting)**								
Incremental benefit	Deaths	22% (-8-49)	22% (3-49)	22% (3-49)	22% (6-41)	20% (3-49)	28% (-2-42)	33% (-8-40)
	Clinical cases	33% (-2-53)	33% (-1-53)	31% (-1-53)	31% (1-52%)	34% (-1-53)	35% (-1-53)	34% (-2-56)
**Cost-effectiveness (ICER per DALY averted.)**								
$2 a dose	6-9 months 4th dose	$42 ($18-298)	$38 ($18-97)	$37 ($18-97)	$31.5 ($18-40)	$64 ($48-97)	$87.5 ($53-126)	$130 ($62-188)
	6-9 months	$45 ($16-312)	$35 ($16-112)	$36 ($18-112)	$26 ($18-45)	$56.5 ($54-112)	$76.5 ($60-135)	$116 ($70-204)
$5 a dose	6-9 months 4th dose	$96 ($48-728)	$87 ($48-244)	$82 ($49-244)	$73.5 ($49-96)	$158 ($105-244)	$214 ($120-312)	$316 ($143-462)
	6-9 months	$84 ($44-763)	$80 ($44-279)	$78.5 ($49-279)	$65 ($49-82)	$139 ($117-279)	$189 ($130-334)	$283 ($159-500)

$10 a dose	6-9 months 4th dose	$197 ($99-1447)	$154 ($99-487)	$160 ($100-487)	$140 ($100-197)	$315 ($200-487)	$426 ($232-622)	$626.5 ($278-919)
	6-9 months	$155 ($90-1514)	$147 ($90-556)	$144.5 ($99-556)	$128.5 ($99-155)	$277.5 ($219-556)	$376.5 ($246-665)	$562.5 ($306-995)
**Cost-effectiveness (ICER per clinical case averted.)**								
$2 a dose	6-9 months 4th dose	$18 ($6-115)	$10 ($6-93)	$13.5 ($6-33)	$9.5 ($6-20)	$26.5 ($18-33)	$37.5 ($21-43)	$61.5 ($27-68)
	6-9 months	$16 ($7-138)	$13 ($7-88)	$13 ($7-38)	$11 ($7-16)	$24 ($22-38)	$34 ($26-53)	$58.5 ($32-81)
$5 a dose	6-9 months 4th dose	$42 ($16-281)	$25 ($16-222)	$32.5 ($16-80)	$19 ($16-49)	$65.5 ($40-80)	$92.5 ($46-108)	$151 ($62-167)
	6-9 months	$40 ($18-337)	$30 ($18-211)	$32 ($18-94)	$21.5 ($18-40)	$60 ($45-94)	$84 ($55-131)	$143 ($71-198)
$10 a dose	6-9 months 4th dose	$84 ($28-558)	$51 ($28-437)	$63 ($29-159)	$36.5 ($29-97)	$131 ($75-159)	$184 ($89-215)	$299.5 ($120-333)
	6-9 months 4th dose	$80 ($31-669)	$61 ($31-415)	$64 ($32-187)	$41 ($32-80)	$119.5 ($84-187)	$167 ($103-260)	$284.5 ($137-394)

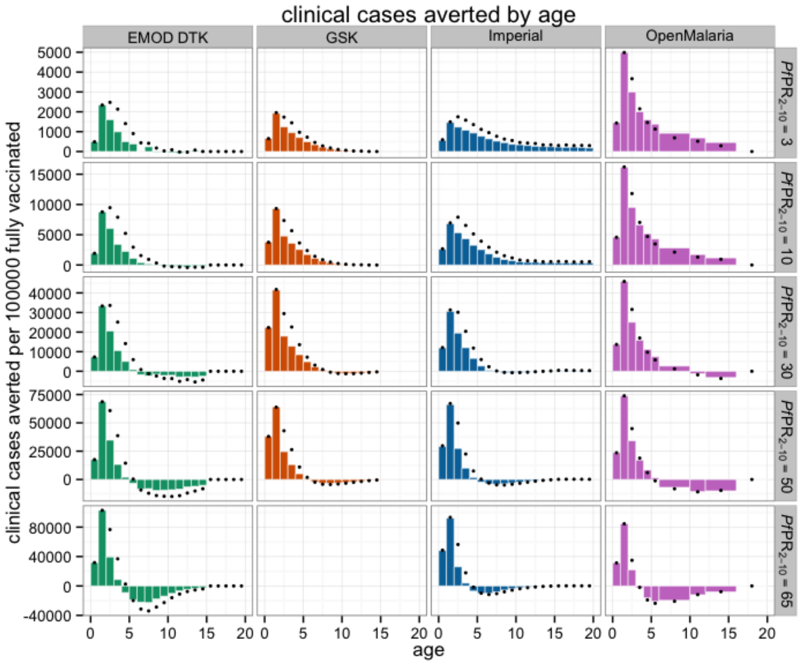

Figure S4.1: Clinical cases averted per 100,000 fully vaccinated children stratified by age group and *Pf*PR$_{2\text{-}10}$. Rows indicate parasite prevalence intensity, columns and colour modelling groups (green EMOD DTK, orange GSK, blue Imperial, purple OpenMalaria). Bars show events averted during 15 years of use of RTS,S within a 6-9 month immunisation schedule; black dots indicated events averted by the same schedule but administration of an additional 4th dose at 27 months. Note the y-axis for each prevalence row are at different scales.

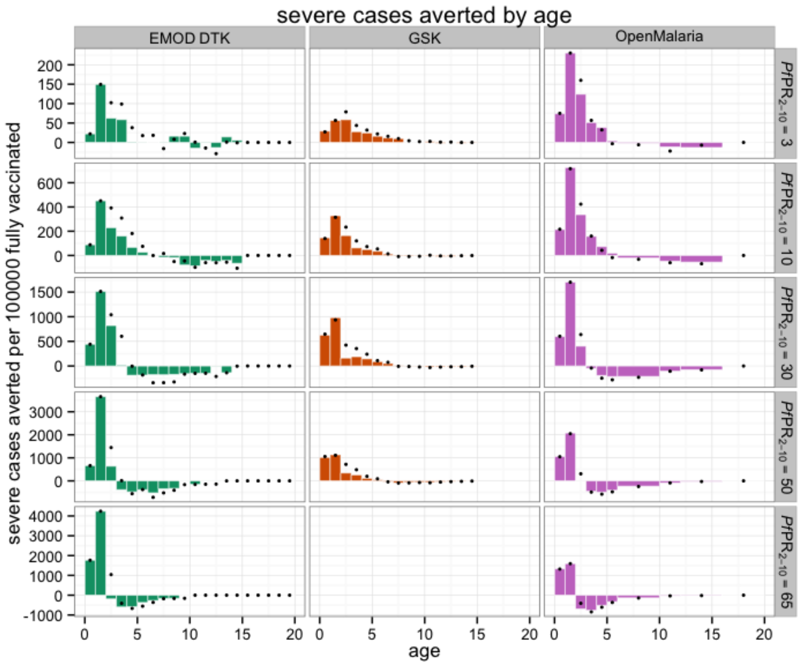

Figure S4.2: Severe cases averted per 100,000 fully vaccinated children stratified by age group and *Pf*PR$_{2\text{-}10}$. Rows indicate parasite prevalence intensity, columns and colour modelling groups (green EMOD DTK, orange GSK, blue Imperial, purple OpenMalaria). Bars show events averted during 15 years of use of RTS,S within a 6-9 month immunisation schedule; black dots indicated events averted by the same schedule but administration of an additional 4th dose at 27 months. Note the y-axis for each prevalence row are at different scales.

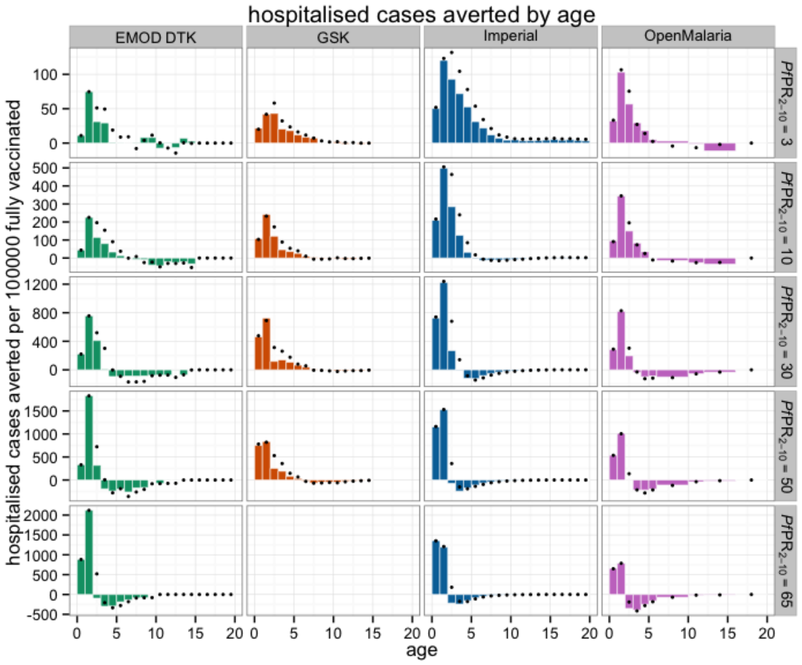

Figure S4.3: Hospitalised cases averted per 100,000 fully vaccinated children stratified by age group and *Pf*PR$_{2\text{-}10}$. Rows indicate parasite prevalence intensity, columns and colour modelling groups (green EMOD DTK, orange GSK, blue Imperial, purple OpenMalaria). Bars show events averted during 15 years of use of RTS,S within a 6-9 month immunisation schedule; black dots indicated events averted by the same schedule but administration of an additional 4th dose at 27 months. Note the y-axis for each prevalence row are at different scales.

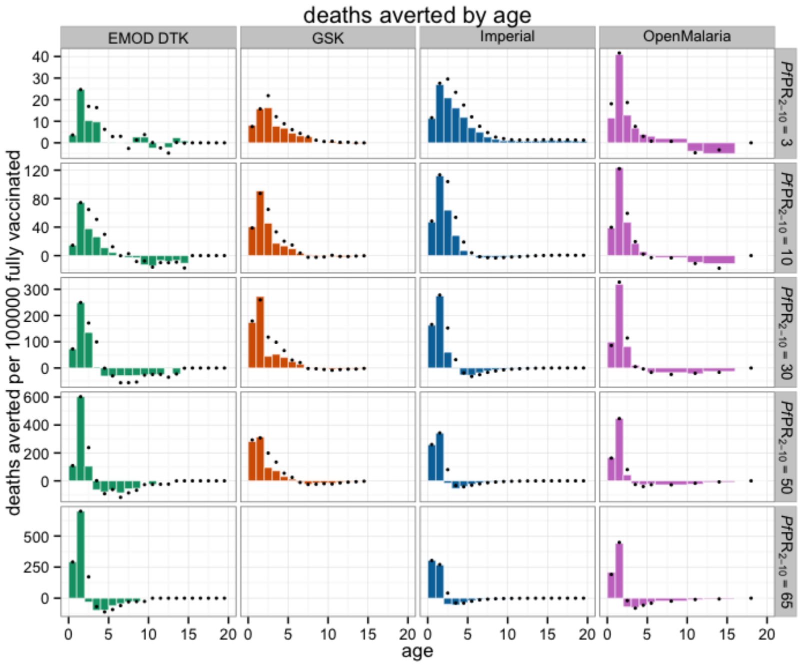

Figure S4.4: Deaths averted per 100,000 fully vaccinated children stratified by age group and *Pf*PR$_{2\text{-}10}$. Rows indicate parasite prevalence intensity, columns and colour modelling groups (green EMOD DTK, orange GSK, blue Imperial, purple OpenMalaria). Bars show events averted during 15 years of use of RTS,S within a 6-9 month immunisation schedule; black dots indicated events averted by the same schedule but administration of an additional 4th dose at 27 months. Note the y-axis for each prevalence row are at different scales.

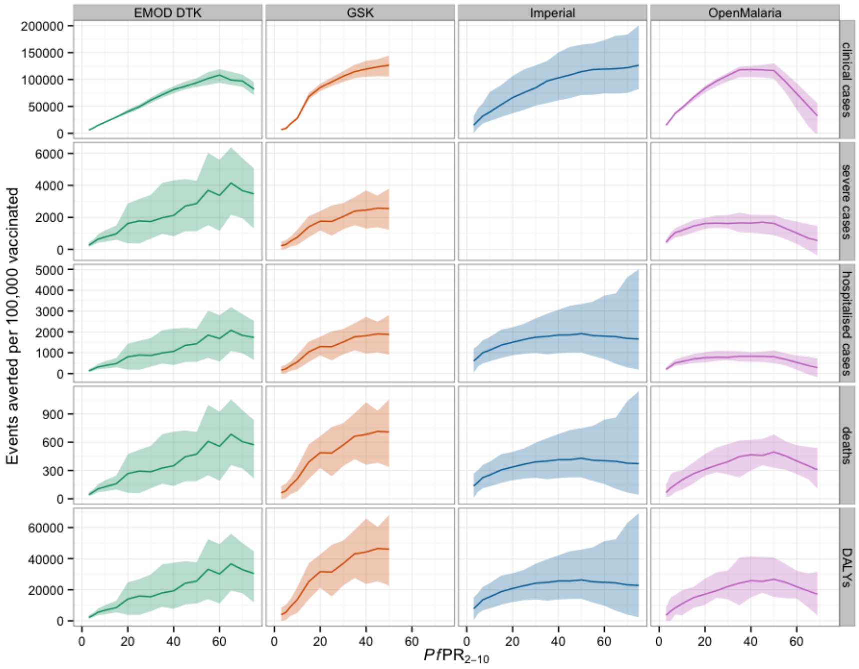

Figure S4.5: The cumulative number of either clinical cases, severe cases, hospitalised cases, death or DALYs averted per 100,000 fully vaccinated children within 15 years after the start of RTS,S vaccination in a 6 to 9 months schedule with a 4th dose (results without 4th dose not shown).

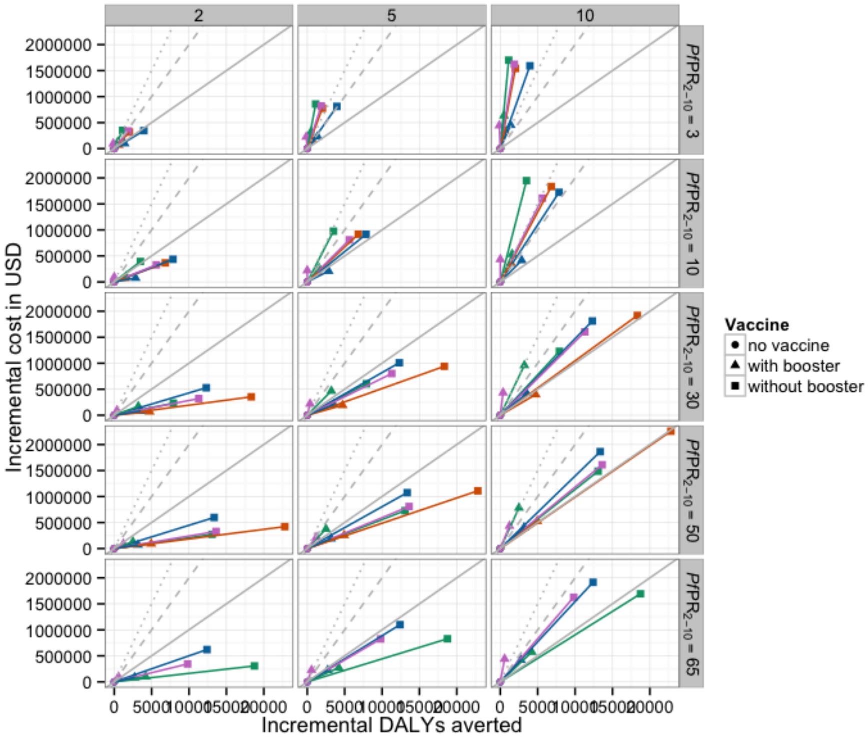

Figure S4.6: Cost effective planes comparing the net program costs of RTS,S with the DALYs averted. Showing total net incremental costs and DALYs averted for use of RTS,S within a 6 to 9months schedule over routine malaria control and incremental costs and DALYs averted of RTS,S with and without 4th dose are shown for different transmission settings and vaccine prices. Grey reference lines represent ratios of 100, 200 and 300 USD per DALY averted. Colour indicates group (green EMOD DTK, orange GSK, blue Imperial, purple OpenMalaria)

5 Sensitivity analysis of RTS,S cost-effectiveness

This section of the Appendix provides details of a sensitivity analysis of RTS,S cost-effectiveness in generic transmission settings. One modelling group- Swiss TPH - assessed the impact of uncertainty around the vaccine properties, health systems and economic parameters on the cost-effectiveness of the vaccine in a series of one-way sensitivity analyses. Since the costing approach was standardised across models, the variation in these factors is expected to be mirrored for the other models.Immunization coverage and access to malaria case management ranges were obtained by scaling the baseline values up and down 25% up to the highest/ lowest values simulated. For vaccine properties best fit values were used in the baseline simulations, low and high posteriors were used for the upper and lower ranges. Additionally estimates of vaccine cost-effectiveness were produced under the worst- and best case assumptions of vaccine properties. Parameter values and ranges are detailed in Table S5.1.

5.1 Harmonization assumptions

Methodology to estimate vaccine efficacy against clinical malaria are detailed in section 2 of Supplementary Appendix. Predictions reported here are based on best-fit vaccine efficacy profiles as estimated by Swiss TPH and detailed in Figure 3 in the main text. The model is harmonized along all other inputs including exposure, demographics, immunization coverage, and costs (see Table 1 and sections 1, 3, 4 in Supplementary Appendix).

5.2 Sensitivity analysis

ICERs were produced for each transmission setting and vaccination schedule by varying each of the parameters detailed in Table S5.1 while holding all other inputs at baseline values. Sensitivity 1-6 and 8-10 in Table S5.1 are one-way sensitivity analyses, where as sensitivity 7 is a scenario analysis on vaccine properties, with the worst case, and best case vaccine properties based on half-life and initial efficacy against infection. The direction and broad magnitude of changes in ICERs were similar for the two deployment modalities. For sake of brevity only estimates for 4-dose schedule are presented. Table S5.2 presents outcomes of one-way sensitivity in terms of cost per DALY averted (cumulative ICER 15 years after program implementation). Figure S5.1 summarizes these results as tornado plots: the cost per DALY averted over the 15-year time horizon at most doubles from the baseline estimate when considering a range of factors including lower vaccination coverage, lower estimates of vaccine efficacy and higher vaccine price, with the greatest impact due to a price increase from $5 to $10.

Table S5.1: List of parameters and ranges varied in one-way sensitivity analysis

Sensitivity	Parameter	Baseline Value	Low Value	High Value
1	Access[a]	45%	33.75%	56.25%
2	Immunization coverage	90%	67.5%	100%
4	Initial Efficacy against infection[b]	92%	75%	99%
5	Efficacy 4th dose	49.1%	32%	69%
6	Half-life[c]	7.32 months	6 months	10.2 months
7	Profile[c]	Efficacy 92%, half-life 7.32 months	Efficacy 75%, half-life 6 months	Efficacy 99%, half-life 10.2 months
8	Vaccine price per dose	$5	$2	$10
9	Discount rate	3%	0%	10%
10	Horizon	15	5	10

[a] Access refers to proportion of fevers that are treated effectively (cured parasitaemia) by the routine case management regardless of source of treatment. [b] Efficacy refers to initial efficacy against infection with lower and upper limits taken from the posterior distributions of initial efficacy from OpenMalaria (Table S2.1), with the lower value (75%) roughly representing representing 80% of baseline. [c] Weibull, biphasic decay.

Table S5.2: OpenMalaria one-way sensitivity of RTS,S cost-effectiveness as ICER per DALY averted by transmission level. The cumulative cost per DALY averted within 15 years after the start of vaccination with RTS,S in a 6 to 9 months schedule with a 4th dose administered 18 months after the third dose (USD 2013).

		***Pf*PR$_{2-10}$= 3**			***Pf*PR$_{2-10}$=10**			***Pf*PR$_{2-10}$=30**			***Pf*PR$_{2-10}$=50**			***Pf*PR$_{2-10}$=65**		
**No**	**Parameter**	**Baseline Value**	**Low Value**	**High Value**	**Baseline Value**	**Low Value**	**High Value**	**Baseline Value**	**Low Value**	**High Value**	**Baseline Value**	**Low Value**	**High Value**	**Baseline Value**	**Low Value**	**High Value**
1	Access[a]	$602	$461	$602	$183	$141	$183	$87	$74	$87	$69	$65	$69	$101	$96	$101
2	Immunization coverage	$602	$602	$669	$183	$183	$203	$87	$87	$97	$69	$69	$77	$101	$101	$112
4	Initial Efficacy[b]	$602	$625	$572	$183	$221	$176	$87	$105	$82	$69	$89	$63	$101	$124	$91
5	Efficacy 4th dose	$602	$636	$531	$183	$207	$161	$87	$96	$78	$69	$74	$64	$101	$106	$94
6	Half-life[c]	$602	$645	$516	$183	$196	$154	$87	$93	$74	$69	$74	$59	$101	$107	$81
7	Profile	$602	$699	$434	$183	$263	$127	$87	$123	$63	$69	$107	$50	$101	$159	$71
8	Profile, coverage	$602	$699	$483	$183	$263	$141	$87	$123	$70	$69	$107	$56	$101	$159	$79
9	Vaccine price per dose	$602	$250	$1'189	$183	$75	$363	$87	$35	$174	$69	$28	$138	$101	$42	$199
10	Discount rate	$602	$734	$410	$183	$223	$124	$87	$107	$60	$69	$85	$47	$101	$124	$68
11	Horizon	$602	$663	$574	$183	$197	$182	$87	$100	$90	$69	$77	$69	$101	$87	$93

[a] Access refers to proportion of fevers that are treated effectively (cured parasitaemia) by the routine case management regardless of source of treatment. [b] Efficacy refers to initial efficacy against infection. [c] Weibull, biphasic decay.

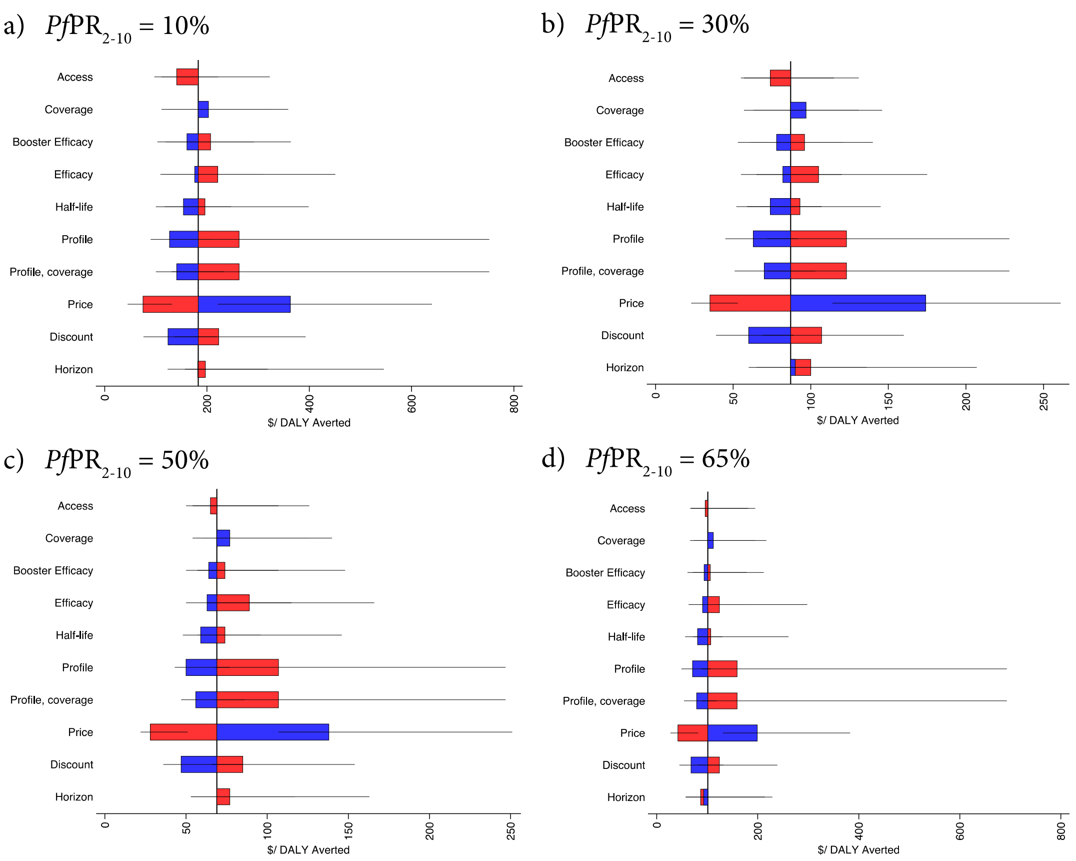

Figure S5.1: OpenMalaria one-way sensitivity and scenario analysis of RTS,S cost-effectiveness. Results show cost per DALY averted (USD, 2013) for 6-9 month schedule with 4 doses by transmission level (a) *Pf*PR$_{2\text{-}10}$=10%, (b) *Pf*PR$_{2\text{-}10}$=30%, (c) *Pf*PR$_{2\text{-}10}$=50%, (d) *Pf*PR$_{2\text{-}10}$=65%.

6 References

1. Carneiro I, Roca-Feltrer A, Griffin JT, et al. Age-patterns of malaria vary with severity, transmission intensity and seasonality in sub-Saharan Africa: a systematic review and pooled analysis. *PLoS One* 2010; **5**: e8988-.
2. Hay SI, Guerra CA, Tatem AJ, Noor AM, Snow R. The global distribution and population at risk of malaria: past, present, and future. *Lancet Infect Dis* 2004; **4**: 327-36.
3. Eckhoff PA. A malaria transmission-directed model of mosquito life cycle and ecology. *Malar J* 2011; **10**: 303.
4. Eckhoff P. P. falciparum infection durations and infectiousness are shaped by antigenic variation and innate and adaptive host immunity in a mathematical model. *PLoS One* 2012; **7**(9): e44950.
5. Ross A, Maire N, Molineaux L, Smith T. An epidemiologic model of severe morbidity and mortality caused by Plasmodium falciparum. *Am J Trop Med Hyg* 2006; **75**: 63-73.
6. Eckhoff PA. Malaria parasite diversity and transmission intensity affect development of parasitological immunity in a mathematical model. *Malar J* 2012; **11**(419): 10-1186.
7. Wenger EA, Eckhoff PA. A mathematical model of the impact of present and future malaria vaccines. *Malar J* 2013; **12**(126): 10-1186.
8. Eckhoff P. Mathematical models of within-host and transmission dynamics to determine effects of malaria interventions in a variety of transmission settings. *Am J Trop Med Hyg* 2013; **88**(5): 817-27.
9. McCarthy KA, Wenger EA, Huynh GH, Eckhoff PA. Calibration of an intrahost malaria model and parameter ensemble evaluation of a pre-erythrocytic vaccine. *Malar J* 2015; **14**(1): 6.
10. Griffin JT, Hollingsworth TD, Okell LC, et al. Reducing Plasmodium falciparum malaria transmission in Africa: a model-based evaluation of intervention strategies. *PLoS Med* 2010; **7**: e1000324.
11. White MT, Griffin JT, Churcher TS, Ferguson NM, Basanez MG, Ghani AC. Modelling the impact of vector control interventions on Anopheles gambiae population dynamics. *Parasites and Vectors* 2011; **4**: 153-.
12. Griffin JT, Ferguson NM, Ghani AC. Estimates of the changing age-burden of Plasmodium falciparum malaria disease in sub-Saharan Africa. *Nat Commun* 2014; **5**: 3136.
13. Griffin JT, Hollingsworth TD, Reyburn H, Drakeley CJ, Riley EM, Ghani AC. Gradual acquisition of immunity to severe malaria with increasing exposure. *Proceedings of the Royal Society B: Biological Sciences* 2015; **282**(1801): 20142657.
14. Smith T, Killeen GF, Maire N, et al. Mathematical modeling of the impact of malaria vaccines on the clinical epidemiology and natural history of Plasmodium falciparum malaria: Overview. *Am J Trop Med Hyg* 2006; **75**: 1-10.
15. Chitnis N, Hardy D, Smith T. A Periodically-Forced Mathematical Model for the Seasonal Dynamics of Malaria in Mosquitoes. *Bull Math Biol* 2012; **74**(5): 1098-124.
16. Sauboin C, Van Vlaenderen I, Van Bellinghen LA, Standaert B. Cost-effectiveness of RTS,S malaria vaccine candidate in children estimated using a Markov cohort model and Phase 3 trial results 3rd International Conference of the African Health Economics and Policy Association; 2014; 2014.
17. Smith T, Maire N, Dietz K, et al. Relationship between the entomologic inoculation rate and the force of infection for Plasmodium falciparum malaria. *Am J Trop Med Hyg* 2006; **75**: 11-8.
18. Smith T, Ross A, Maire N, et al. Ensemble modeling of the likely public health impact of a pre-erythrocytic malaria vaccine. *PLoS Med* 2012; **9**(1): 44.
19. Maire N, Smith T, Ross A, Owusu-Agyei S, Dietz K, Molineaux L. A model for natural immunity to asexual blood stages of Plasmodium falciparum malaria in endemic areas. *Am J Trop Med Hyg* 2006; **75**: 19-31.
20. Ross A, Killeen GF, Smith T. Relationships of host infectivity to mosquitoes and asexual parasite density in Plasmodium falciparum *Am J Trop Med Hyg* 2006; **75 (Suppl 2)**: 32-7.
21. Smith T, Ross A, Maire N, Rogier C, Trape JF, Molineaux L. An epidemiologic model of the incidence of acute illness in Plasmodium falciparum malaria. *Am J Trop Med Hyg* 2006; **75**: 56-62.

22. Penny MA, Maire N, Bever C, et al. Distributions of malaria exposure in endemic countries in Africa considering country levels of effective treatment. *Malaria Journal (in press)* 2015.
23. Aponte JJ, Menendez C, Schellenberg D, et al. Age interactions in the development of naturally acquired immunity to Plasmodium falciparum and its clinical presentation. *PLoS Med* 2007; **4**(7): e242.
24. Sauboin C, Van Vlaenderen I, Van Bellinghen LA, Standaert B. Public health impact of RTS,S malaria vaccine candidate in children estimated using a Markov cohort model and Phase 3 trial results. 6th MIM Pan-African Malaria Conference; 2013; 2013.
25. White MT, Bejon P, Olotu A, et al. A combined analysis of immunogenicity, antibody kinetics and vaccine efficacy from phase 2 trials of the RTS,S malaria vaccine. *BMC Med* 2014; **12**(1): 117.
26. Eckhoff PA, Bever CA, Gerardin J, Wenger EA. Fun with maths: exploring implications of mathematical models for malaria eradication. *Malar J* 2014; **13**(1): 486.
27. Ross A, Smith T. The effect of malaria transmission intensity on neonatal mortality in endemic areas. *Am J Trop Med Hyg* 2006; **75 (Suppl 2)**: 74-81.
28. Nunes JK, Cardenas V, Loucq C, et al. Modeling the public health impact of malaria vaccines for developers and policymakers. *BMC Infect Dis* 2013; **13**: 295-.
29. Trape JF, Rogier C. Combating malaria morbidity and mortality by reducing transmission. *Parasitol Today* 1996; **12**: 236-40.
30. Kitua A, Smith T, Alonso PL, et al. Plasmodium falciparum malaria in the first year of life in an area of intense and perennial transmission. *Trop Med Int Health* 1996; **1**: 475-84.
31. World Health Organization. Annex 2. Estimated and reported cases and deaths, 2006. Geneva: World Health Organization; 2008: 141-4.
32. Reyburn H, Mbatia R, Drakeley C, et al. Association of transmission intensity and age with clinical manifestations and case-fatality of severe Plasmodium falciparum malaria. *J Am Med Assoc* 2005; **293**: 1461-70.
33. Marsh K, Snow R. Malaria transmission and morbidity. *Parassitologia* 1999; **41**: 241-6.
34. Rowe A, Rowe SY, Snow RW, et al. The burden of malaria mortality among African children in the year 2000. *Int J Epidemiol* 2006; **35**(3): 691-704.
35. Snow R, Omumbo J, Lowe B, et al. Relation between severe malaria morbidity in children and level of Plasmodium falciparum transmission in Africa [see comments]. *Lancet* 1997; **349**: 1650-4.
36. Okiro EA, Al-Taiar A, Reyburn H, Idro R, Berkley JA, Snow RW. Age patterns of severe paediatric malaria and their relationship to Plasmodium falciparum transmission intensity. *Malar J* 2009; **8**(1): 4.
37. Bhatt S, Weiss DJ, Cameron E, et al. The effect of malaria control on Plasmodium falciparum in Africa between 2000 and 2015. *Nature* 2015; **advance online publication**.
38. Galactionova K, Tediosi F, Savigny Dd, Smith TA, Tanner M. Effective coverage and systems effectiveness for malaria case management in Sub-Saharan African countries. *PLoS One* 2015; **10**(5).
39. Hay SI, Guerra CA, Gething PW, et al. A world malaria map: Plasmodium falciparum endemicity in 2007. *PLoS Med* 2009; **6**: e1000048-.
40. Chitnis N, Schapira A, Smith T, Steketee R. Comparing the effectiveness of malaria vector-control interventions through a mathematical model. *Am J Trop Med Hyg* 2010; **83**: 230-40.
41. World Health Organization. World Malaria Report 2012. Geneva: World Health Organisation, 2013.
42. White MT, Verity R, Griffin JT, et al. Immunogenicity of the RTS,S/AS01 malaria vaccine over time and implications for duration of vaccine efficacy: analysis of data from a phase 3 randomised controlled trial. *Lancet Infect Dis* 2015; **doi:10.1016/S1473-3099(15)00239-X**.
43. Penny MA, Galactionova K, Tarantino M, Tanner M, Smith TA. The public health impact of malaria vaccine RTS, S in malaria endemic Africa: country-specific predictions using 18 month follow-up Phase III data and simulation models. *BMC Med* 2015; **13**(1): 170.
44. Open Malaria decay functions. https://github.com/SwissTPH/openmalaria/wiki/ModelDecayFunctions#configuration (accessed 1/6/2015 2015).
45. Hutton G, Tediosi F. The costs of introducing a malaria vaccine through the expanded program on immunization in Tanzania. *Am J Trop Med Hyg* 2006; **75**: 119-30.

46. Maire N, Shillcutt SD, Walker DG, Tediosi F, Smith TA. Cost-effectiveness of the introduction of a pre-erythrocytic malaria vaccine into the expanded program on immunization in sub-Saharan Africa: analysis of uncertainties using a stochastic individual-based simulation model of Plasmodium falciparum malaria. *Value Health* 2011; **14**: 1028-38.
47. Murray CJL, Lopez AD. The global burden of disease: a comprehensive assessment of mortality and disability from diseases, injuries, and risk factors in 1990 and projected to 2020. Harvard: Harvard University Press; 1996.
48. INDEPTH Network. Model Life Tables for Sub-Saharan Africa. Aldershot, England: Ashgate; 2004.
49. Murray CJ, Ezzati M, Flaxman AD, et al. GBD 2010: design, definitions, and metrics. *Lancet* 2012; **380**: 2063-6.
50. Greenwood BM, David PH, Otoo-Forbes LN, et al. Mortality and morbidity from malaria after stopping malaria chemoprophylaxis. *Trans R Soc Trop Med Hyg* 1995; **89**: 629-33.
51. Pemberton-Ross P, Smith TA, Hodel EM, Kay K, Penny MA. Age-shifting in malaria incidence as a result of induced immunological deficit: a simulation study. *Malar J* 2015; **14**(1): 287.
52. Ghani AC, Sutherland CJ, Riley EM, et al. Loss of population levels of immunity to malaria as a result of exposure-reducing interventions: consequences for interpretation of disease trends. *PLoS One* 2009; **4**(2): e4383.
53. Gosling RD, Ghani AC, Deen JL, von Seidlein L, Greenwood BM, Chandramohan D. Can changes in malaria transmission intensity explain prolonged protection and contribute to high protective efficacy of intermittent preventive treatment for malaria in infants? *Malar J* 2008; **7**: 54.
54. RTSS Clinical Trials Partnership. Efficacy and safety of RTS,S/AS01 malaria vaccine with or without a booster dose in infants and children in Africa: final results of a phase 3, individually randomised, controlled trial. *Lancet* 2015; **386**(9988): 31-45.
